# Machine learning and deep learning approaches in IoT

**DOI:** 10.7717/peerj-cs.1204

**Published:** 2023-02-06

**Authors:** Abqa Javed, Muhammad Awais, Muhammad Shoaib, Khaldoon S. Khurshid, Mahmoud Othman

**Affiliations:** 1 Department of Computer Science, University of Engineering and Technology, Lahore, Punjab, Pakistan; 2 Computer Science Department, Future University in Egypt, New Cairo, Egypt

**Keywords:** IoT (Internet of Things), IoMT (Internet of Medical Things), IoV (Internet of Vehicles), IPS (Intrusion Prevention System), Machine learning, Deep learning

## Abstract

The internet is a booming sector for exchanging information because of all the gadgets in today’s world. Attacks on Internet of Things (IoT) devices are alarming as these devices evolve. The two primary areas of the IoT that should be secure in terms of authentication, authorization, and data privacy are the IoMT (Internet of Medical Things) and the IoV (Internet of Vehicles). IoMT and IoV devices monitor real-time healthcare and traffic trends to protect an individual’s life. With the proliferation of these devices comes a rise in security assaults and threats, necessitating the deployment of an IPS (intrusion prevention system) for these systems. As a result, machine learning and deep learning technologies are utilized to identify and control security in IoMT and IoV devices. This research study aims to investigate the research fields of current IoT security research trends. Papers about the domain were searched, and the top 50 papers were selected. In addition, research objectives are specified concerning the problem, which leads to research questions. After evaluating the associated research, data is retrieved from digital archives. Furthermore, based on the findings of this SLR, a taxonomy of IoT subdomains has been given. This article also identifies the difficult areas and suggests ideas for further research in the IoT.

## Introduction

The Internet of Things (IoT) is the network of objects embedded with sensors and software to exchange information with other devices over the internet (Hassan et al., 2019). The immense usage of IoT devices (Atzori, Iera & Morabito, 2017), including wearable devices, smartphones, and sensors, has been adopted (Da Xu, He & Li, 2014) to help humans to achieve their daily life goals (Almiani et al., 2020). Since the last decade, a great proliferation has been observed in IoT devices, from eight billion to 41 billion in the next five years (Ahmad & Alsmadi, 2021) and their users from five billion to 27 billion. Due to the tremendous rate of IoT devices, subdomains of IoT are being emerged, such as the Internet of Medical Things (IoMT) and Internet of Vehicles (IoV) (Quasim et al., 2019). IoMT and IoV are directly related to humans (Sullivan, 2022), so these are emerging fields in IoT. Authentication, privacy, access control, confidentiality, and unauthorized access to computing devices are major challenges for IoT devices in the internet era (Rizvi et al., 2018).

With the increasing number of internet devices and users (Sharma & Liu, 2020), there could be numerous problems and challenges which need to be addressed for security and privacy issues (Sha et al., 2018). The security of IoT devices without passwords is a significant security concern. Most IoT devices are used without a password or with only a simple password. Hackers can simply gain access to these devices and abuse them. These flaws expose data and allow unauthorized people to get access to IoT devices. Infrastructure and privacy of networked IoT devices are required to protect against attacks since these vulnerabilities cause network capacity to be exceeded (Ahmad & Alsmadi, 2021). However, attacks against IoMT devices can endanger valuable human lives; on the other hand, IoV requires safe and secure networks to function properly. In case of an attack on these lifesaving technologies, we need to develop countermeasures to cope with these challenges.

Researchers proposed rule-based systems to overcome the security issues in the IoT domain (Feng et al., 2018). However, these rule-based systems do not work properly with the latest security attacks (Makkar et al., 2021). Machine learning has emerged as a rising field in the network security domain. Systems that can learn from past data are a good measure to ensure security (Bagaa et al., 2020). Although these systems have been used to secure the network security issues (Vaiyapuri, Binbusayyis & Varadarajan, 2021), they require specific hardware and software.

When dealing with IoT devices, they do not have high-end computing. Researchers have been actively working on lightweight software that does not require expensive, complex hardware (Boutros-Saikali, Saikali & Abou Naoum, 2018). To review the recent literature about the mentioned topic, researchers have conducted research to assist the practitioners in studying the core concepts of IoT security. Some of those researchers have only worked on the SLR of machine learning-based approaches, such as Xiao et al. (2018) discussed machine learning for IoT (Cui et al., 2018), and other authors only focused on the deep learning-based approach (Zikria et al., 2020; Tiwari et al., 2019). The authors worked on these topics are limited to only IoV (Yang et al., 2017), IoMT (Papaioannou et al., 2020), and IPSs (Oke et al., 2018). The previous SLR ignored the security issues such as authentication, confidentiality, authorization, and privacy (Tahsien, Karimipour & Spachos, 2020).

This research addresses the mentioned shortcomings of the previous systematic review to fill this gap. This study’s systematic literature review is conducted based on developed research questions.

To the best of our knowledge, no prior researchers have worked on the systematic review of IoT security issues in its subdomains, including IoMT, IoT, and IoH. Additionally, the prior works are limited to the traditional security issues such as intrusion detection and prevention problems (Priyan & Devi, 2019). Furthermore, the IoT devices do not contain powerful hardware, so processing the attack information could be challenging. Therefore, a detailed systematic review of these security attacks in resource-constrained IoT domains and their sub-fields is necessary. Additionally, this survey can help the early-stage researchers and practitioners to know more about these emerging fields. In Table 1, we have compared our work with the prior research studies based on the following three dimensions, including IoV security, IoMT, and IPS for IoT devices. Based on the systematic literature review, 50 papers have been selected that cover the basic criteria. This survey covers only those papers related to IoT security, IoMT, IoT, and IPS. Papers older than 2016 are not included in this survey. Moreover, papers that are not covered the main security issues such as authentication, authorization, and privacy are not included in the survey. The selected papers are evaluated qualitatively and empirically in different aspects.

**Table 1:** Comparison with related work.

**IoMT**	**Focus of study**	**Survey approach**	**Year**	**Quality assessment score**	**Explored survey perspectives**		
					**Security critical application**	**IPS**	**Combine ML and DL approach**
Bai et al. (2021)	IoT in the healthcare domain	SLR	2021	3	IoMT	×	Machine learning
Abbasi et al. (2021)	Internet of vehicles	SLR	2021	×	IoV	×	×
Patel, Qassim & Wills (2010)	Intrusion prevention system	Informal	2010	×	IoV	3	Machine learning
This survey	ML and DL approaches in IoT security	SLR	2021	3	IoMT and IoV	×	ML and DL

The novelty of this systematic literature review is that it covers all the existing literature related to IoT security in the domains of IoMT, IoV, and IPS. Moreover, the research covers the security aspects in terms of privacy, authentication, and authorization in all defined domains of IoT. According to the query string, no identified survey covers all these dimensions.

This article is organized as follows: Section II covers the existing literature survey and provides the path to the current SLR of the article. Section III presents the methodology adapted to conduct a good survey with research questions and objectives. Section IV covers the answers to these research questions, and Section V presents the taxonomy of the domain. The last section, VI, covers the conclusion of the article.

## Literature Review

Bai et al. (2021) published a systematic review on the security issues in the healthcare domain. They focused on the security and provenance issues for the Internet of Medical Things. As per the authors, no prior work was done on the security issues for the IoT in the medical domain. Their work only focuses on the security issues for the healthcare domain, and they reviewed the existing security issues from 2011 to 2020. They selected sixty-nine papers from five repositories related to IoT applications in the healthcare domain.

Additionally, the present work only focused on a single dimension of IoT regarding security and provenance. They do not address the security issues, particularly device authentication, authorization, and data privacy. Moreover, this article focused on machine learning and deep learning techniques to overcome the security issues in IoMT, IoV, and IPS.

Another study conducted by Abbasi et al. (2021) focused on the applications and challenges of the internet of vehicles. The authors categorized the services and applications of the internet of vehicles. Their work only focuses on the application and service issues for the IoV domain, and they reviewed the existing literature from 2010 to 2019. The selected papers were from six digital repositories related to IoT applications in vehicular networks. They also discussed some of the challenges and open issues in the current domain. The major focus of this research was to study the services and applications of the internet of vehicles. However, this study did not address a major component of the internet of vehicles, *i.e.,* security issues. According to the author, the security and accuracy of the systems are very important in deploying these systems. However, the current study ignors this dimension of this work. An insecure system might not work in a real-time environment, and it will always be open to new security attacks. Therefore, security issues in the recent internet of vehicles domain must be addressed to deploy the newer systems in real-time. This article did not perform the quality assessment on the selected papers and ignored those that worked with machine learning and deep learning techniques to secure IoMT and IoV. Furthermore, only the intrusion detection systems are discussed, not the intrusion prevention system to secure systems before the attack.

Patel, Qassim & Wills (2010) presented the intrusion prevention system covering security-related issues. The authors categorized the intrusion detection and prevention system according to the security perspective. This survey deals with both the intrusion detection and prevention systems, which are helpful for the users to cover the basic security issues. IPS is working with the security tools such as firewalls and malware filters. This review only considers the related literature from 2000 to 2010. The selected papers discussed that as the number of Internet-related devices increases, security issues are also raised. Internet-connected devices are affected by different security issues such as malware intrusion, authorization, and violation of private data. As the IoT devices are not mature enough to handle the security, there is a need to implement an advanced intelligent system for security that maintains data privacy in all aspects and avoids unknown attacks. However, the current study has not mentioned the dimensions of the work. Therefore, a lightweight system should be implemented to resolve the authorization and authentication issues in IPS. The current study discussed the machine learning techniques and ignored the proper quality assessment criteria for the selected studies.

Selected studies discuss machine learning and deep learning techniques to secure the devices from attacks. The studies mentioned in Table 1 have shortcomings as they have only focused on a single subdomain of IoT and have presented literature on its security constraints (Cui et al., 2018). However, as these security constraints vary from field to field, and thus an opportunity exists to synthesize the existing work into a single study to perform a comparative analysis. In this regard, the novelty of our study includes IoMT, IoV, and IPS subdomains for the complete SLR. According to the defined research questions, papers are selected from 2016 to 2021.

## Research Methodology

Systematic literature review guidelines (Bai et al., 2021) are followed in this review. Three main stages are included in this review according to the research protocol that is: planning, conducting, and reviewing the data. Search protocol is described after the finalization of research questions. These research questions are helpful to search the related review data and avoid the biasness in the selected studies.

### Review plan

**Figure 1:**
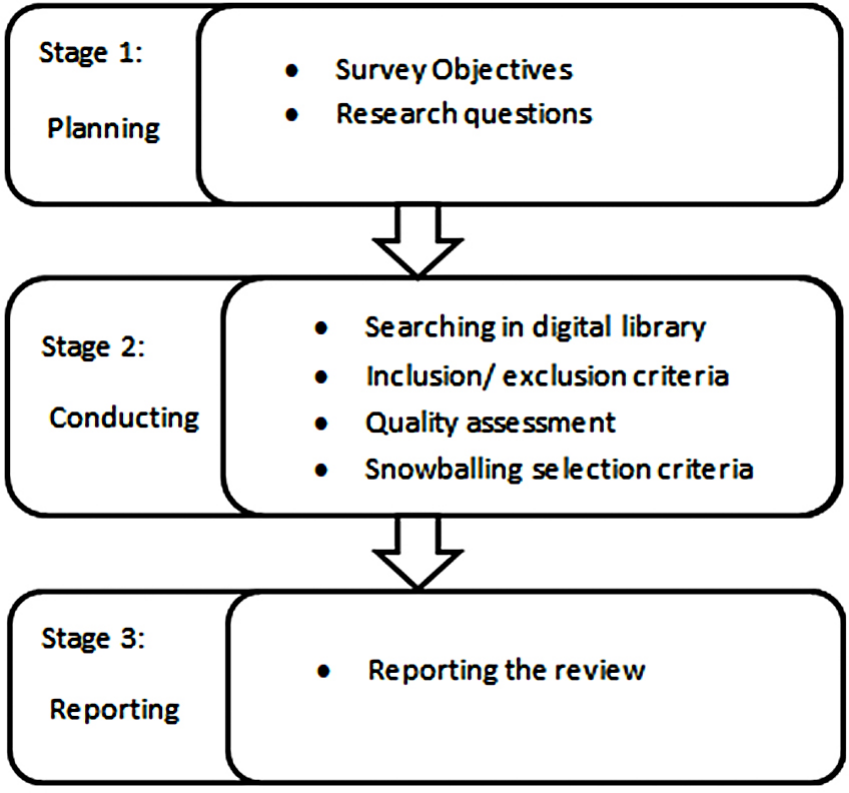
Research strategy.

**Figure 2:**
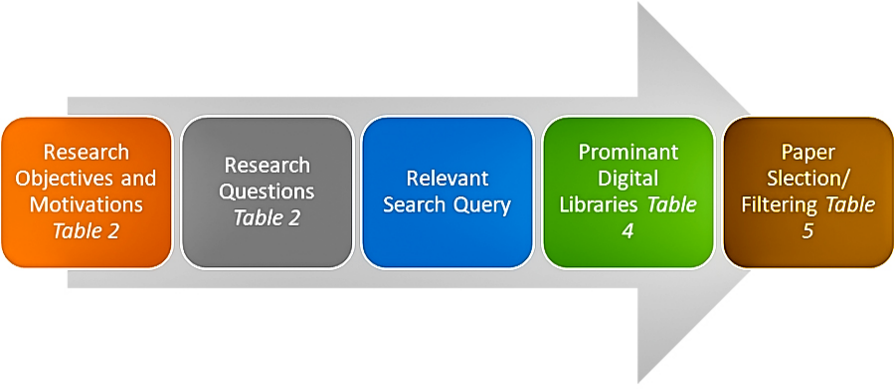
Search strategy.

Figures 1 and 2 show the methodology that defines the research process for the classification scheme, relevant publications, and publications mapping criteria. A search strategy has been implemented to find all the related data of IoT. A complete systematic approach is used to select the relevant studies without biases. In this review, the structured process has been followed that involved:

• Research objectives
• Research questions
• Organizing search repositories
• Selection studies
• Screening results
• Data extraction
• Results
• Review report finalization

To achieve the objectives of the above-defined review plan, see the RQs in Table 2:

• The RQ1 identifies and explores different high-level databases that have been published in the literature on IoT-related smart devices security by using machine learning and deep learning. These answers might help choose the best venues from the highest priority platforms.
• RQ2 helps assist the primary study conducted within the last five years, which discusses the implementation of secure systems in IoT.
• RQ3 deals with the basic methods to implement authorization, authentication, and privacy in IoMT, IoV, and IPS environments.
• The objective of RQ4 is to implement machine learning and deep learning techniques to cover all the security issues faced by the IoMT.

### Review conduct

In this systematic literature review, we have extracted the most relevant data from the selected digital databases. Furthermore, the predefined inclusion/exclusion criteria select the papers from the repositories. Moreover, the quality assessment is added to enhance the paper selection approach. After that, the most important papers are extracted from the existing literature by implementing snowballing techniques.

**Table 2:** Research questions.

**RQ**	**Research question statement**	**Objectives and motivations**
RQ1:	Which are relevant publication channels for IoT research?	To identify
		• high-quality publication venues for IoT research.
		• IoT research publications during 2016-2021.
RQ2:	What are the current challenges in different IoT types regarding implementing security measures?	To understand the different security requirements and protect IoT devices from massive malicious attacks.
RQ3:	What are some of the authorization and authentication methods used for general IoT security purposes?	To identify the key methods that are used for authorization and authentication in IoT networks, including
		• IoMT
		• IoV
		• IPS
RQ4:	How can we implement or utilize lightweight ML-based security methods on resource-constrained IoMT devices?	To identify the recommended machine learning techniques to protect IoMT devices from attacks.

#### Automated search in digital repositories

Systematic search is implemented to extract the related data from the available online repositories and filter the irrelevant information. Moreover, manual and automatic search techniques have been applied while exploring the search terms. Different digital libraries were visited during this process, and only those repositories have been selected that are searched from our search process and commonly accessed literature survey. Those public venues are selected that are related to SLR. Google Scholar also added the venue that even accessed the data from the indirect venues. Therefore, the following digital venues are covered almost all the relevant searches selected as a primary source for automatic search:

• Google Scholar (https://scholar.google.com/)
• HEC Digital Library (http://www.digitallibrary.edu.pk/)
• ACM Digital Library (http://dl.acm.org)
• IEEE eXplore (http://ieeexplore.ieee.org)
• ScienceDirect (https://www.sciencedirect.com)

Manual search is implemented to collect more related literature on IoT machine learning techniques and their related domain. The extracted information will provide a limited search of the related data, so it is specified according to the given conditions:

• Primary keywords are selected based on the research questions
• Identify the secondary keywords that were used as additional keywords
• The search string is developed by adding the “AND” and “OR” Boolean operators

Primary keywords are chosen as key identifiers to search the IoT data. Secondary or additional keywords are added with the primary keyword to search the related data. Boolean operators, keywords, and wildcards have been added to develop the final search query.

**Figure 3:**
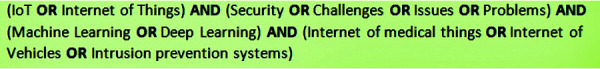
Search query.

**Figure 4:**
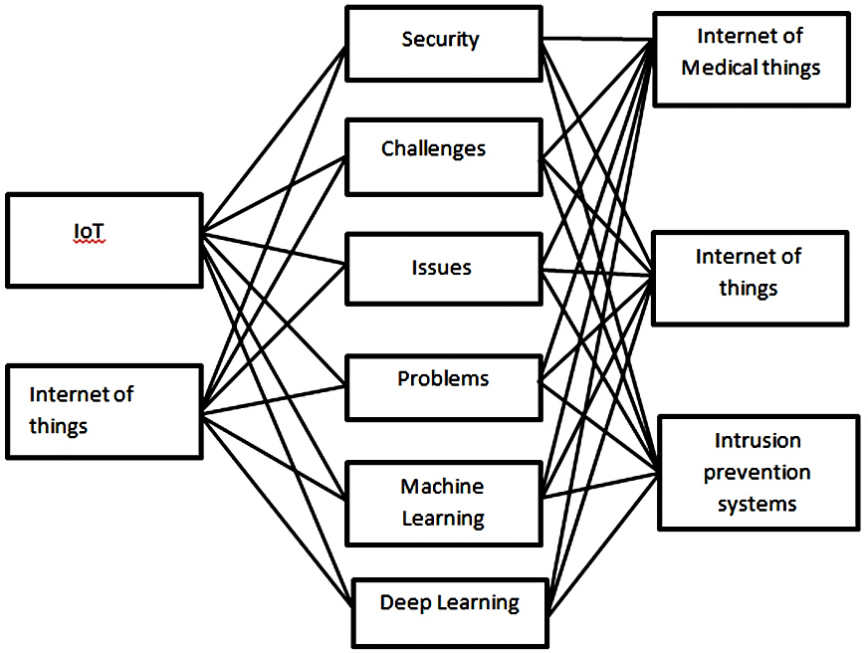
Search string describe work included in our knowledge base.

Figure 3 defines the search query that is restrictive to appear during the initial search process. The query is unable to search the final string data. The final string query is too restrictive and searches only the related articles on Google scholar and other defined repositories. Moreover, after implementing the search query, related articles are selected that fulfill the defined criteria of the papers. Additionally, selecting studies with string queries is very effective compared to traditional systems. Figure 4 depicts the visualization of all possible combinations of the query string.

#### Selection based on Inclusion/Exclusion Criteria

#### Inclusion criteria

• The included paper must have the IoT as a central topic.
• The paper must target the research questions.
• Selected papers must be published in the SJR index journal.
• Conference papers must be published in the top conferences.
• Paper explores challenges, issues, and shortcomings of IoT devices.
• The paper must discuss the IoMT, IoV, and IPS.
• Papers must discuss the machine learning and deep learning methods to solve the IoT problems.

#### Exclusion criteria

• Papers are excluded that are not written in the English language.
• Exclude papers that do not discuss any RQ.
• Exclude papers that are published before 2016.
• Exclude duplicate papers.
• Add the most recent version of the paper.

#### Selection based on quality assessment

The selection of the papers is based on the quality assessment, which is the most important step in conducting any review. Quality assessment is done to enhance the quality of the paper. As the primary study papers vary in the design, different tools such as qualitative and quantitative methods (Bai et al., 2021), and Abbasi et al. (2021) are used to perform the QA in the review. QA of our study carries out by the three authors, and each study is scored based on the defined criteria:

1. The paper published in Impact Factor journal awarded 2, otherwise 1.
2. Paper covers more than 3 IoT security issues award 2, if it discusses anyone IoT security issue award 1, otherwise 0.
3. Paper has citation award 1, else than 0.
4. If a paper has research gap award 1, define the problem award 2; otherwise, 0.
5. Paper discusses the evaluation of the research paper award 2 if results are given award 1, otherwise 0.
6. The conclusion is given of the paper award 1, otherwise 0.

The overall score of the questions is 10. The papers having scored more than 6 are included to finalize the results. Table 3 shows the possible scores for the Journals and Conferences with the grading 0 to 4.

#### Selection based on Snowballing

**Figure 5:**
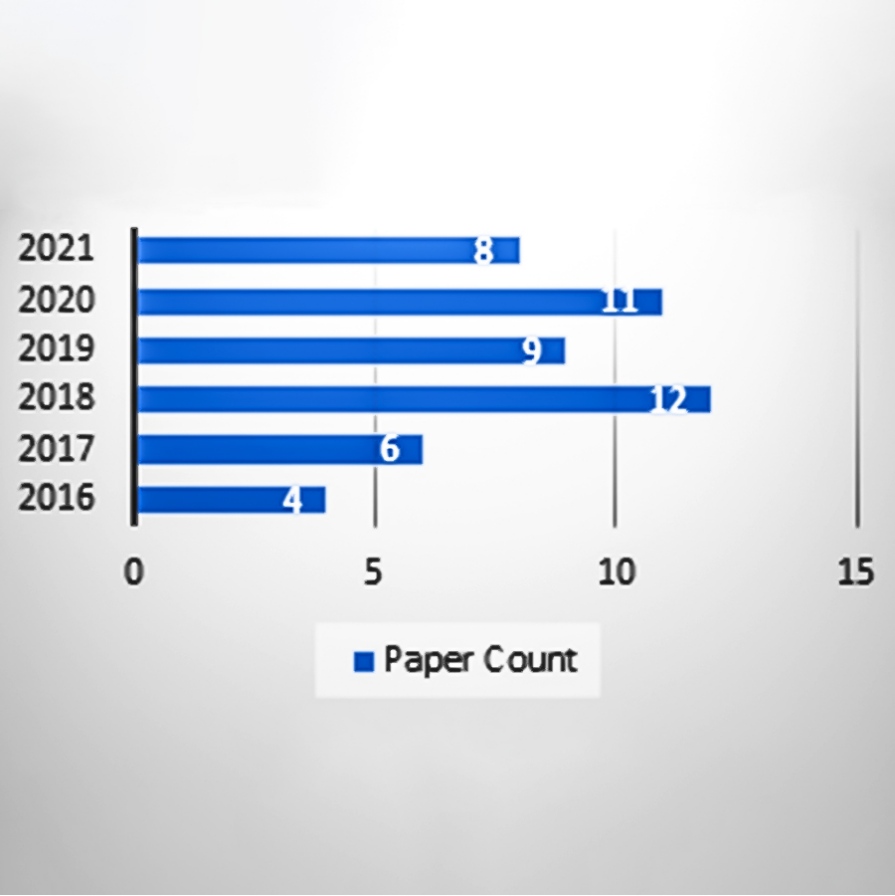
Paper count according to years.

After performing the quality assessment technique, snowballing is implemented on Bai et al. (2021) reference list to finalize the extracted papers. Only those papers are selected through snowballing that fulfill the criteria of inclusion/exclusion. The papers are found by implementing search query on different digital libraries that are defined in Table 4. searching. The inclusion/exclusion of the paper is decided after reading the abstract of the paper and then reading the other part of the paper. Figure 5 shows that total 50 papers are extracted by filtering.

### Review report

The final selected papers are inspected thoroughly and selected; the 50 papers are based on the search query and fulfill inclusion/exclusion criteria. Overview of the selected papers from the above query is mentioned in Table 4. Papers are excluded less than the four pages and filter papers according to the following parameters: since 2016, title, introduction, abstract, and conclusion. Finally, the papers are extracted with full articles. The paper count of per year is defined in the Fig. 5.

**Table 3:** Possible scores for Journals and Conferences.

**Pblication source**	**+4**	**+3**	**+2**	**+1**	**+0**
Journals	Q1	Q2	Q3	Q4	No JCR Ranking
Conferences, Workshops, Symposia	CORE A*	CORE A	CORE B	CORE C	Not in CORE Ranking


**Table 4:** Search strategy for selected repositories.

**Digital library**	**Search query**	**Applied filter**
Google Scholar	(IoT OR Internet of Things) AND (Security OR Challenges OR Issues OR Problems) AND (Machine Learning OR Deep Learning) AND (Internet of medical things OR Internet of Vehicles OR Intrusion prevention systems)	Since 2016
HEC Digital Library	(IoT OR Internet of Things) AND (Security OR Challenges OR Issues OR Problems) AND (Machine Learning OR Deep Learning) AND (Internet of medical things OR Internet of Vehicles OR Intrusion prevention systems)	Since 2016
ACM Digital Library	[[All: iot] OR [All: internet of things]] AND [[All: security] OR [All: challenges] OR [All: issues] OR [All: problems]] AND [[All: machine learning] OR [All: deep learning]] AND [[All: internet of medical things] OR [All: internet of vehicles] OR [All: intrusion prevention systems]]	Since 2016
IEEE eXplore	(IoT OR Internet of Things) AND (Security OR Challenges OR Issues OR Problems) AND (Machine Learning OR Deep Learning) AND (Internet of medical things OR Internet of Vehicles OR Intrusion prevention systems)	Since 2016
ScienceDirect	(IoT OR Internet of Things) AND (Security OR Challenges OR Issues OR Problems) AND (Machine Learning OR Deep Learning) AND (Internet of medical things OR Internet of Vehicles OR Intrusion prevention systems)	Since 2016

**Figure 6:**
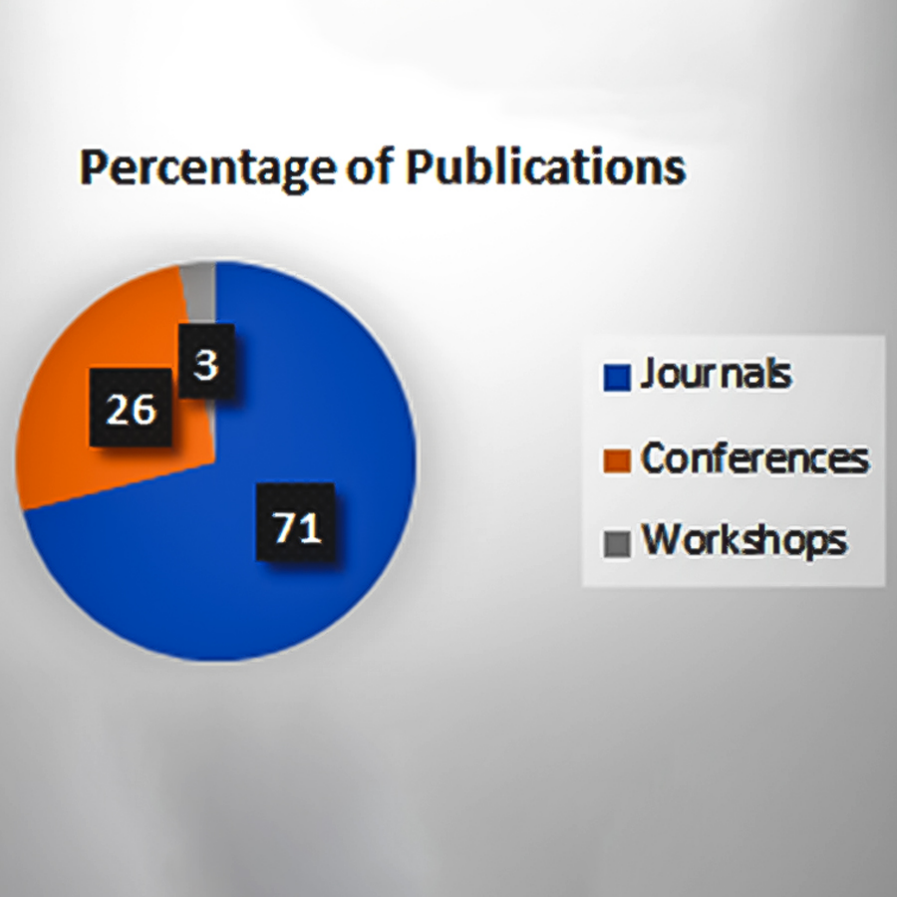
Percentage of publication types.

Figure 6 shows that most of the journal papers are added in this review paper, and the reports are skipped as they are not fulfilling the inclusion/exclusion criteria.

**Figure 7:**
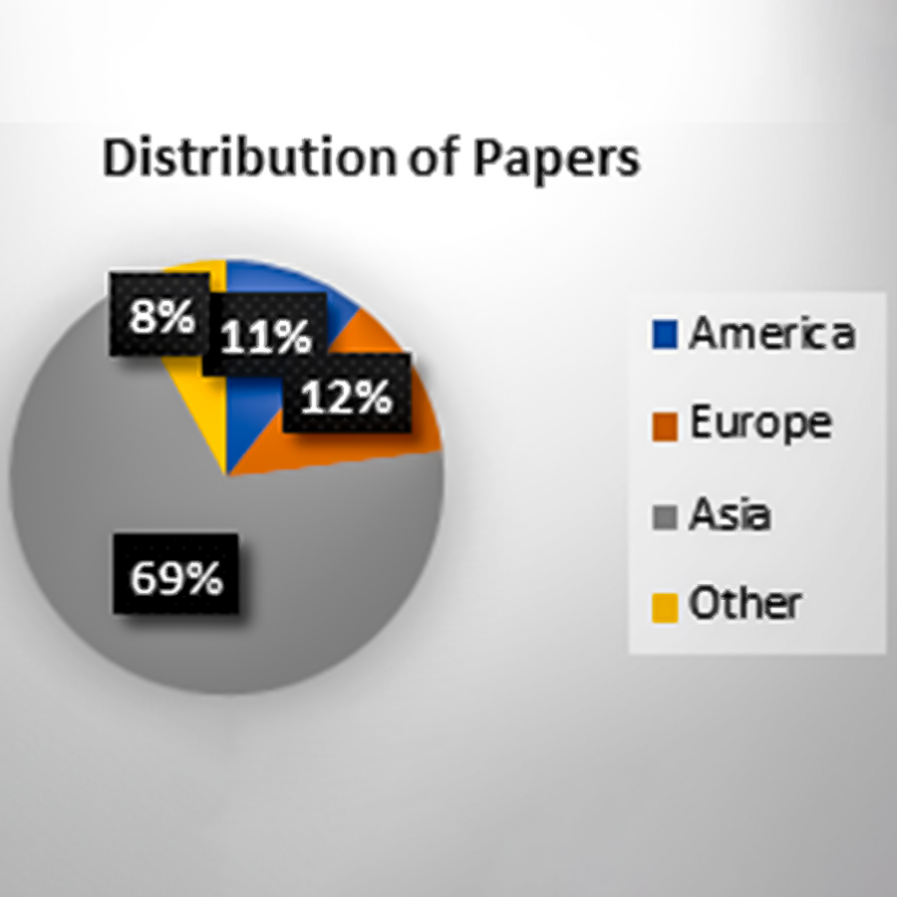
Geographical area distributions.

Figure 7 shows that the selected papers are from a different geographical areas, and most of them belong to the different states of America.

## Assessment and Discussion of Research Questions

The research questions are evaluated from the selected 50 papers extracted from a systematic literature review.

### Which are relevant publication channels for IoT research?

IoT security is still a challenging domain in research due to the growth of IoT devices which causes security threats. There is a need to identify the proper publication tools and venues to access the relevant data to solve these security issues in the IoT domain. Moreover, this section presents knowledge base publication sources, types, and publication channels.

After the inspection phase, a maximum of eight publications are selected from the IEEE eXplore and one from the ACM journal, as mentioned in Table 5. These publications are considered the world’s largest professional publishing source. Table 6 presents all the publication channels from where the papers are selected for the current SLR. Table 7 discussed the contribution and proposed solutions that are provided by different authors related to current studies.

Moreover, Table 8 presents the quality score of the each study which determines the classification of the studies for the systematic literature review. These studies are classified based on empirical search, research type and methodology. Quality assessment score are used to categorized the studies that are included in the paper. These empirical studies are further classified such as surveys, evaluation studies, primary search and experimental search. On the basis of these classifications, research taxonomy is defined in later sections. The codes are assigned including IoV and IoM for Internet of Vehicle and Internet of Medical Things respectively. Future research is clearly defined the path for the new researcher to explore more relevant studies.

**Table 5:** Query string generated selection and result phase.

**Phase**	**Selection**	**Selection criteria**	**Google scholar**	**HEC digital library**	**ACM digital library**	**IEEE eXplore**	**Science direct**	**Total papers**
1	Search	Keywords (Fig. 3)	48700	632	425	16322	32500	98579
2	Filtering	Since 2016	17500	368	370	6803	2100	27141
3	Filtering	Title	168	24	160	128	150	630
4	Filtering	Abstract	140	19	15	30	10	214
5	Filtering	Introduction and conclusion	58	15	3	15	7	98
6	Inspection	Full article	23	13	1	8	5	50

**Table 6:** Publication source.

**Publication source**	**Channel**	**References**	**No.**
Computer networks	Journal	Hassan et al. (2019)	1
CHOICE	Conference	Quasim et al. (2019)	1
IEEE Internet of Things Journal	Journal	Sharma & Liu (2020), Makkar et al. (2021)	2
Future Generation Computer Systems	Journal	Sha et al. (2018)	1
IEEE Access	Journal	Bagaa et al. (2020)	1
International Journal of Advanced Computer Science and Applications	Journal	Vaiyapuri, Binbusayyis & Varadarajan (2021)	1
Journal of Communications and Information Networks	Journal	Yang et al. (2017)	1
Journal of Network and Computer Applications	journal	Tahsien, Karimipour & Spachos (2020)	1
Internet of Things	journal	Ahmad & Alsmadi (2021)	1
International Journal of Machine Learning and Cybernetics	journal	Haseeb et al. (2021)	1
International Journal of Advanced Intelligence Paradigms	journal	Priyan & Devi (2019)	1
Journal of Software: Evolution and Process	journal	Bai et al. (2021)	1
International Journal of Communication Systems	journal	Abbasi et al. (2021)	1
Information Management and Computer Security	Conference	Patel, Qassim & Wills (2010)	1

**Table 7:** Solution proposed by selected studies.

**Study**	**Contribution**	**Approach**
Haseeb et al. (2021)	Predict the consumption of network resources and improve the delivery of sensors data over the internet.	The centralized-based software defines network (SDN) architecture to overcome network threats among deployed sensors with nominal management cost.
Nandy et al. (2021)	This paper identifies the attacks while data transmission over the network and identifies the efficiency of health data with higher accuracy.	An Empirical Intelligent Agent (EIA) based on a unique Swarm-Neural Network (Swarm-NN) method is designed to secure data on the network.
Chaudhry et al. (2021)	The secure system presented a device-to-device (D2D) communication framework for suitable communication in the IoMT environment.	D2DAC-IoMT model is proposed to enhance the system’s performance regarding security and improve efficiency.
Ali, Hassan & Saeed (2021)	IoV edge computing is presented for authentication and authorization.	Machine learning and reinforcement learning-based approaches are used in mobile edge computing for the IoV environment.
Sikarwar & Das (2021)	A new architecture for IoV is designed to cover the ITS and challenges of IoV to secure communication and provide authentication.	Vehicular ad-hoc networks (VANETs) focused on the various applications for intelligent transportation systems for traffic management and safe driving.
Chandre, Mahalle & Shinde (2021)	Full proof intrusion prevention framework presented to overcome the malicious attacks over the network in IoT devices.	A deep learning-based model is implemented for intrusion prevention, which gives the best accuracy compared with the artwork’s state.

**Table 8:** Classification of studies.

Ref	Classification					Quality assessment						
	Publication channel	Year	Research type	Empirical type	Methodology/Task	A	B	C	D	E	F	Total score
Sha et al. (2018)	Journal	2018	primary	Experimental	Security	2	1	1	0	1	1	6
Boutros-Saikali, Saikali & Abou Naoum (2018)	conference	2018	Primary	Experimental	IoMT	1	1	1	2	2	1	8
Papaioannou et al. (2020)	Journal	2020	Primary	Experimental	IoMT	2	2	1	1	1	1	8
Oke et al. (2018)	Journal	2018	Primary	Experimental	IPS	2	1	1	1	3	1	9
Hatzivasilis et al. (2019)	Conference	2019	Primary	Experimental	IoMT	1	2	1	1	1	1	7
Kotenko, Saenko & Branitskiy (2018)	Journal	2018	Primary	Experimental	ML-based approach	2	2	1	1	2	1	9
Holbrook & Alamaniotis (2019)	Conference	2019	Primary	Experimental	Dl based approach	1	2	0	1	2	1	7
Khamparia et al. (2020)	Journal	2020	Primary	Experimental	Dl based approach	2	2	1	2	1	1	9
Sumathi & Karthikeyan (2021)	Journal	2021	Primary	Experimental	Dl based approach	2	1	1	2	3	1	10
Tama & Rhee (2017)	Journal	2017	Primary	Experimental	Dl based approach	1	1	1	1	2	1	7
Sumathi & Pugalendhi (2021)	Journal	2021	Primary	Experimental	ML and DL based approach	2	2	1	1	2	1	9
Al-Hawawreh & Sitnikova (2019)	Conference	2019	Primary	Experimental	DL based approach	0	2	1	2	2	1	8
Yavuz, Devrim & Ensar (2018)	Journal	2018	Primary	Experimental	Attack detection	1	2	1	1	2	1	8
Kasongo & Sun (2020)	Journal	2020	Primary	Experimental	Dl based approach	1	2	1	1	3	1	9
Zhou et al. (2018)	Conference	2018	Primary	Experimental	DL based approach	0	1	1	1	2	1	6
Almiani et al. (2020)	Journal	2020	Primary	Experimental	DL based approach	1	1	1	1	2	1	7
Tuan et al. (2020)	Journal	2020	Primary	Experimental	Deep Learning models	2	1	1	1	2	1	8
Min et al. (2018)	Journal	2018	Primary	Experimental	ML-based approach	2	1	1	1	2	1	8
Kasyoka, Kimwele & Mbandu Angolo (2020)	Journal	2021	Primary	Experimental	DL based approach	2	1	2	1	1	1	8
Khan et al. (2022)	Journal	2022	Primary	Experimental	IoMT	2	1	2	1	1	1	8
Sayeed et al. (2019)	Journal	2019	Primary	Experimental	IoMT	1	2	1	1	1	1	7
Kumar et al. (2019)	Journal	2019	Primary	Experimental	IoV	2	1	0	1	1	1	6
Uprety, Rawat & Li (2021)	Conference	2021	Primary	Experimental	IoV	1	2	1	1	1	1	7
Mahmood (2020)	Journal	2020	Primary	Experimental	IoV	2	2	1	1	1	1	8
Anbalagan et al. (2021)	Journal	2021	Primary	Experimental	ML Based approach	1	2	1	1	2	1	8
Verma & Ranga (2020)	Journal	2020	Primary	Experimental	IPS	2	2	1	1	2	1	9
Zhang et al. (2020)	Journal	2020	Primary	Experimental	DL Based approach	1	1	1	1	1	1	6
Chatterjee et al. (2019)	Journal	2021	Primary	Experimental	ML Based approach	1	0	1	1	2	1	6
Mawgoud, Karadawy & Tawfik (2019)	Journal	2019	Primary	Experimental	ML Based approach	1	2	1	1	1	1	7
Vajar et al. (2021)	Conference	2021	Primary	Experimental	IoMT	1	1	1	1	1	1	6
Pirbhulal et al. (2019)	Conference	2019	Primary	Experimental	ML-based approach	1	2	1	1	1	1	7
Aljumaie et al. (2021)	Journal	2021	Primary	Experimental	IoMT	2	2	1	1	2	1	9
Thapa & Camtepe (2021)	Journal	2021	Primary	Experimental	IoMT	2	2	1	1	1	1	8
Newaz et al. (2019)	Conference	2019	Primary	Experimental	IoMT	1	1	1	1	1	1	6
Lv et al. (2020)	Journal	2020	Primary	Experimental	DL based approach	2	2	1	1	2	1	9
Das et al. (2018)	Conference	2018	Primary	Experimental	DL based approach	1	1	1	1	2	1	7
Li et al. (2021)	Journal	2021	Primary	Experimental	DL based approach	2	1	1	1	2	1	8
Sai et al. (2021)	Conference	2021	Primary	Experimental	DL based approach	1	1	1	1	1	1	6
Guan et al. (2019)	Journal	2019	Primary	Experimental	IoMT	2	2	1	1	1	1	8
Raj & Madiajagan (2021)	Journal	2021	Primary	Experimental	IoMT	2	2	1	1	2	1	9
Hameed et al. (2021)	Journal	2021	Primary	Experimental	IoMT	2	2	1	1	1	1	8
Ali, Hassan & Saeed (2021)	Journal	2021	Primary	Experimental	IoV	2	1	1	1	2	1	8
Rajapkar, Binnar & Kazi (2020)	Conference	2020	Primary	Experimental	IPS	1	1	1	1	1	1	6

### What are the current challenges in different IoT types regarding implementing security measures?

IoMT and IoV are the major areas in the IoT domain discussed regarding security, as these are sensitive to human life. In recent years, security measures have been implemented in these areas, such as authentication, authorization, and privacy (Tahsien, Karimipour & Spachos, 2020). Some current challenges discussed in Table 9 are faced with implementing the security measures. Moreover, the existing literature has not considered the authentication, authorization, and privacy of data in the IoMT and IoV domain using machine learning and deep learning techniques. Machine learning and deep learning algorithms are implemented to overcome these challenges to cover security issues (Cui et al., 2018; Stiawan, Abdullah & Idris, 2010).

**Table 9:** Current challenges in IoT domains.

**Ref.**	**Current challenges**
Tahsien, Karimipour & Spachos (2020)	Privacy, Authentication
Cui et al. (2018)	Security, Identification and Edge computing infrastructure
Ayoubi et al. (2018)	Anomaly detection, Misuse detection
Md. Fadlullah et al. (2017)	Anomaly detection, Auto Encoders
Hammerschmidt et al. (2017)	Malware detection, Interpretability
Hatzivasilis et al. (2019)	Device Security, Connectivity Security, Cloud Security, and Privacy
Bai et al. (2021)	Security and Provenance
Patel, Qassim & Wills (2010)	Intrusion detection and prevention system, encryption, authentication
Chandre, Mahalle & Shinde (2021)	Security and privacy
Sikarwar & Das (2021)	Authentication, security, and privacy
Holbrook & Alamaniotis (2019)	Anomaly detection
Mawgoud, Karadawy & Tawfik (2019)	Privacy
Bhatia, Verma & Sharma (2020)	Encryption, privacy, security
Vajar et al. (2021)	Security and privacy

### What are some of the authorization and authentication methods used for general IoT security purposes?

According to the existing literature, Tama & Rhee (2017), security is the major issue in the IoT domain. Security methods are implemented to secure the IoT in all perspectives, including authentication and authorization (Uprety, Rawat & Li, 2021). A two-way authentication method provides the required security and resists attacks (Mahmood, 2020). If one factor is compromised, the second factor still provides enough security to the IoT system. Elliptic-curve cryptography (ECC) keys are mostly used for one-factor authentication (Bhatia, Verma & Sharma, 2020) as it provides overall lightweight and reliable protection. Moreover, biometric sensors are used as second-factor authentication for everyday use due to their convenient approach (Sikarwar & Das, 2021). Different methods of authentication and authorization are described in Table 10.

**Table 10:** Authorization and Authentication methods for IoT security.

**Ref.**	**Authorization and authentication methods**
Uprety, Rawat & Li (2021)	Federated training
Kumar et al. (2019)	Anti Jammer scheme
Newaz et al. (2019)	IDS embedded in SDN controller
Lv et al. (2020)	Security detection model stacked stack noise encoder and stack autoencoder.
Das et al. (2018)	Incentives fog node, Nash equilibrium solution
Sikarwar & Das (2021)	Biometric sensors
Li et al. (2021)	Encrypt edge data transmission, edge NPU central device
Sai et al. (2021)	TCP/IP, IDS classifier
Guan et al. (2019)	Efficient differentially private data clustering scheme
Hameed et al. (2021)	Hoeffding tree majority class, Hoeffding tree Naïve Bayes, Hoeffding tree Naïve Bayes Adaptive.
Anbalagan et al. (2021)	Memetic-based roadside unit
Mawgoud, Karadawy & Tawfik (2019)	Delimitated anti-jamming protocol
Kasyoka, Kimwele & Mbandu Angolo (2020)	WBANs elliptic curve cryptography
Min et al. (2018)	TR-IDS intrusion detection system
Holbrook & Alamaniotis (2019)	DNN algorithm

### How can we implement or utilize lightweight ML-based security methods on resource-constrained IoMT devices?

Attackers mostly target the integrity and availability of the IoMT systems. AI techniques build detection models to avoid these attacks (Holbrook & Alamaniotis, 2019). Machine learning and deep learning models are used for intrusion detection. When any suspicious activities are detected in the system, then termination of the compromised connection is imposed to diminish the attack. In Table 11, ML-based lightweight methods are described in this study.

**Table 11:** ML-based lightweight methods.

**Ref.**	**Lightweight methods**
Hameed et al. (2021)	Incremental k-nearest neighbor, Hoeffding tree majority class, Hoeffding tree naïve Bayes adaptive
Sharma & Liu (2020)	Naïve Bayes, ensembling with boosting and voting, K-nearest neighbor, support vector machine.
Makkar et al. (2021)	Cognitive spammer framework, fuzzy rule-based classifier
Bagaa et al. (2020)	Network function virtualization, Software-defined networking
Tahsien, Karimipour & Spachos (2020)	Support vector machine, naïve Bayes, k-nearest neighbor, decision tree
Cui et al. (2018)	Naïve Bayes, Decision tree, k-means clustering algorithm
Nandy et al. (2021)	Unsupervised learning algorithms
Ali, Hassan & Saeed (2021)	Markov decision process
Tuan et al. (2020)	Support vector machine, decision tree, Naïve Bayes, Unsupervised learning
Kumar et al. (2019)	CatBoost algorithm, decision tree

## Discussion and Future Direction

This section summarizes the result related to this systematic literature review.

### Taxonomic hierarchy

**Figure 8:**
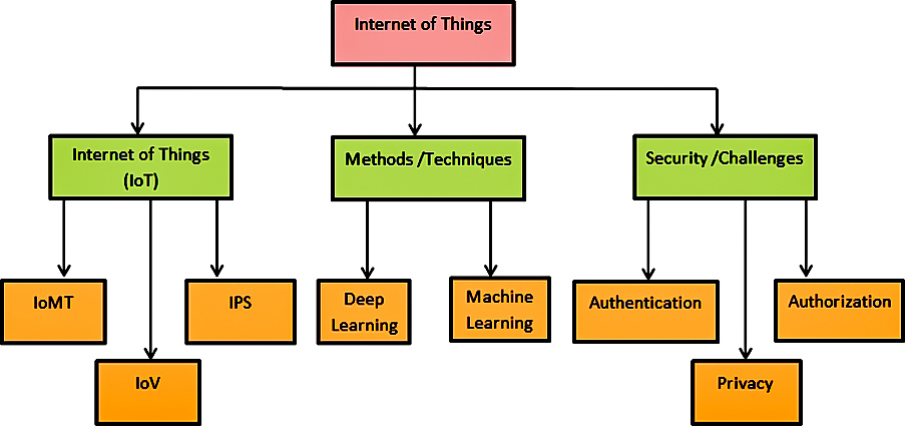
Taxonomic hierarchy.

This systematic literature review aimed to implement the security parameters by selecting the relevant papers and critically reviewing them. We designed the taxonomic hierarchy of selected studies shown in Fig. 8 that are only focused on the security issues of the IoV domain. We have investigated the challenges and developments in different aspects, including high-level features, methods, and security areas. However, these aspects are further divided into sub-domains that show each aspect’s depth and their role in terms of secure devices. Table 12 shows the criteria, evaluation and findings of the papers that are added in the review.

**Table 12:** Criteria, evaluation, and findings of related papers.

**Ref.**	**Criteria**	**Evaluation**	**Findings**
Khamparia et al. (2020)	Combine the big data and machine learning models for Security monitoring.	DBNs are characterized by a graph-like structure, which defines a set of observable and unobservable random variables, changing over time by the transition model.	The model allows detecting multi-step attacks and provides the ability to calculate the probability of how an observable event is anomalous.
Sumathi & Pugalendhi (2021)	Security analytics and solutions	Feature extraction from cervical images is performed using pre-trained CNN models like InceptionV3, VGG19, SqueezeNet, and ResNet50, fed into a dense and fattened layer for normal and abnormal cervical cells classification.	The performance of the proposed IoHT frameworks is evaluated using the standard Pap smear Herlev dataset.
Tama & Rhee (2017)	Distributed denial of services	The performance metrics such as detection accuracy, cost per sample, average delay, packet loss, overhead, packet delivery ratio, and throughput are used for the performance analysis.	Simulation results observed that the DNN Cost minimization algorithm provides a better result in high detection accuracy of 99
Sumathi & Karthikeyan (2021)	analysis of deep learning algorithms	The deep learning artificial neural networks (DLANNs) model is used, which builds a feed-forward multi-layer artificial neural network (ANN) for modeling high-level data abstractions.	IoT datasets show that C4.5 and C5.0 have better accuracy, memory efficiency, and higher processing speeds.
Al-Hawawreh & Sitnikova (2019)	Detection of attacks	Cooja IoT simulator has been utilized to generate high-fidelity attack data within IoT networks ranging from 10 to 1,000 nodes.	Detection of IoT routing attacks with high accuracy and precision is decreased rank, hello-flood, and version number modification attacks.
Yavuz, Devrim & Ensar (2018)	Feature extraction for IPS	The effectiveness and efficiency of the WFEU-FFDNN are studied based on the UNSW-NB15 and the AWID intrusion datasets.	Proposed WFEU-FFDNN has greater accuracy.
Kasongo & Sun (2020)	Machine learning methods for IoT security	Machine learning methods SVM, ANN, NB, DT, and Unsupervised Learning are investigated for Accuracy, False Alarm Rate (FAR), Sensitivity, Specificity, False positive rate (FPR), AUC, and Matthews correlation coefficient (MCC) of datasets.	The performance of the KDD99 dataset has been experimentally shown to be better than the UNBS-NB 15 dataset.
Zhou et al. (2018)	IoT security using Deep learning algorithms	Word embedding, and a text-convolutional neural network (Text-CNN) is applied to extract useful information from payloads.	The sophisticated random forest algorithm is implemented for the final classification.

### General observations and future directions

This systematic literature review studies different machine learning and deep learning methods. We have reviewed an extensive number of studies; another IoT-related security is implemented in the domain of IoMT, IoV, and IPS. Moreover, selected studies were shortlisted considering the defined inclusion/exclusion criteria and quality assessment scoring. In addition, thematic analysis was performed to extract relevancy relations from these selected studies, which are coded.

The codes are selected from the existing literature shows in Table 13, as “IoMT” for the internet of medical things, “IoV” for the internet of the vehicle, and the “IPS” for intrusion prevention systems. After that, papers are selected that worked on machine learning and assigned them the code in the domain of machine learning which is “MM”, “MV”, and “MP”. In the last, papers are categorized according to the deep learning techniques that are “DM”, “DV”, and “DP”. Selected studies were carried out by assessing and analyzing their aims, methodologies, area of discussion, and limitations.

**Table 13:** Coding scheme for SLR.

**Domain**	**Code**	**Sub-Domain abbreviation**
IoT	IoMT	Internet of Medical Things
IoT	IoV	Internet of Vehicle
IoT	IPS	Intrusion prevention system
Machine Learning	MM	Machine learning on the Internet of Medical Things
Machine Learning	MV	Machine learning on the Internet of Vehicle
Machine Learning	MP	Machine learning in intrusion prevention system
Deep Learning	DM	Deep learning on the Internet of Medical Things
Deep Learning	DV	Deep learning on the Internet of Vehicle
Deep Learning	DP	Deep learning in intrusion prevention system

Furthermore, Table 14 defined the codes that are implemented on the selected papers from the defined query strings. All the IoT devices have low computing power, so there is a need to implement a security model covering these embedded devices’ authentication, authorizations, and privacy issues. A lightweight method is found to solve the security issues in the IoMT domain, as mentioned in the RQ4.

**Table 14:** Taxonomy code.

**Ref.**	**Title**	**Taxonomy code**
Boutros-Saikali, Saikali & Abou Naoum (2018)	An IoMT platform to simplify the development of healthcare monitoring applications	IoMT
Papaioannou et al. (2020)	A survey on security threats and countermeasures on the Internet of Medical Things (IoMT)	IoMT
Oke et al. (2018)	Two layers trust-based intrusion prevention system for wireless sensor networks	IPS
Hatzivasilis et al. (2019)	security and privacy for the Internet of Medical Things (IoMT)	IoMT
Nandy et al. (2021)	A machine learning SDN-enabled big data model for IoMT systems	MM
Kotenko, Saenko & Branitskiy (2018)	Framework for mobile Internet of Things security monitoring based on big data processing and machine learning	MM
Khamparia et al. (2020)	Internet of health things-driven deep learning system for detection and classification of cervical cells using transfer learning	MM
Sumathi & Pugalendhi (2021)	Detection of distributed denial of service using deep learning neural network	DP
Tama & Rhee (2017)	Attack classification analysis of IoT network via deep learning approach	DM
Al-Hawawreh & Sitnikova (2019)	Leveraging deep learning models for ransomware detection in the industrial internet of things environment	DV
Yavuz, Devrim & Ensar (2018)	Deep learning for detection of routing attacks in the Internet of Things	DP
Kasongo & Sun (2020)	A deep learning method with wrapper based feature extraction for the wireless intrusion detection system	DP
Almiani et al. (2020)	Deep recurrent neural network for IoT intrusion detection system	DP
Khan et al. (2022)	Explainable simple recurrent units for threat detection on Internet of Medical Things networks	DM
Sayeed et al. (2019)	A machine learning-based fast and accurate seizure detection system in the IoMT	MM
Kumar et al. (2019)	Delimitated anti jammer scheme for the Internet of Vehicle: Machine learning-based security approach	MV
Uprety, Rawat & Li (2021)	Privacy-preserving misbehavior detection in IoV using federated machine learning	MV
Anbalagan et al. (2021)	Machine learning-based efficient and secure RSU placement mechanism for software defined-IoV	MV
Verma & Ranga (2020)	Machine learning-based intrusion detection systems for IoT applications	MP
Chatterjee et al. (2019)	A novel smart healthcare monitoring system using machine learning and the Internet of Things	MM
Mawgoud, Karadawy & Tawfik (2019)	A secure authentication technique on the internet of medical things through machine learning	MM
Pirbhulal et al. (2019)	Towards machine learning-enabled security framework for IoT-based healthcare	MM
Aljumaie et al. (2021)	Modern study on Internet of Medical Things (IOMT) security	IoMT
Newaz et al. (2019)	A machine learning-based security framework for smart healthcare systems	MM
Lv et al. (2020)	Deep-learning-enabled security issues in the Internet of Things	DM
Li et al. (2021)	Deep learning insecurity of Internet of Things	DM
Hameed et al. (2021)	A Hybrid Lightweight System for Early Attack Detection in the IoMT	MM
Ali, Hassan & Saeed (2021)	Machine learning technologies on Internet of Vehicles	MV
Rajapkar, Binnar & Kazi (2020)	Design of intrusion prevention system for OT networks using deep neural networks	DP

IoT domain faces huge challenges in IoMT, IoV, and IPS to security. Uprety, Rawat & Li (2021) investigated the challenges, including weak password protection, insecure interfaces, less data protection, and poor management of IoT devices. The main challenge identified in this article is authorization and authentication problems. Two-way factor analysis techniques are implemented to implement the authorization in IoT. Two-way factor authentications (Mahmood, 2020) are selected as the best security option and avoid attacks. If any security is compromised, then the other one provides essential security. Elliptic-curve cryptography (ECC) keys are used for first-factor authentication in IoMT and other domains of IoT, as they are lightweight and provide reliable protection. Security issues are also raised when the data is delivered over the internet, so constrained application protocols are used to overcome these security issues. Constrained application protocol (Kumar et al., 2019) is like the application protocol used for resource-constrained IoT applications, including IoMT and IoV.

Moreover, some attacks target the availability and integrity of the system, such as stepping-stone attacks. Therefore, the deep neural network is implemented to build the intrusion prevention system. However, important gaps need to be addressed to ensure the IoT devices are secure and not affected by any attacks on critical infrastructures.

The main objective of this SLR is to cover the security issues in the different domains of the internet of things by considering related articles. In order to accomplish the security issues, the hierarchical taxonomy of the selected articles is formulated in Fig. 8. At the top of the hierarchy discussed the internet of things as it is the major concern area. This taxonomy hierarchy shows the broader view of the SLR. It has inspected the different methods and security issues in IoT, including IoMT, IoV, and IPS. Furthermore, machine learning and deep learning methods are defined with the security issues of authorization, authentication, and privacy in the sub-levels to better understand the IoT and its domains.

### Questions for primary study

According to the defined systematic literature review, we carried out the following shortcomings in the existing research.

• What are the other major security issues in IoT subdomains, and which intrusion detection and prevention system exists that covers all the security issues in IoT subdomains?
• Which model can be implemented for the security of IoT devices in all the domains, including IoMT, IoV, IoH, and IoT. Future research requires authentication, vulnerability, condentiality, authorization, and privacy methods.
• Most techniques used for IPS are not provided complete security on complex attacks. Future researchers can develop the intrusion prevention systems for IoT that can be implemented for multiple IoT subdomains to secure devices from all attacks.
• Different security methods are implemented according to the nature of the IoT domain, including a signature group scheme with various limitations. Therefore, Researchers are suggested to implement the security in IoT domains that protect the devices from different attacks to access better results.
• In the current era, heavy models are implemented in IoT devices to deal with complex and dynamic attacks. All the IoT devices have less computing power and cannot tackle this heavy software to overcome the security issues. Therefore, a lightweight method has been required that covers the security issues in the domain of IoT and provides the authentic model to secure these devices from vulnerable attacks.

As the enhancement in IoT, there is a need to implement more security issues to provide a secure IoT environment. Different hardware and software security parameters are implemented to protect the data from interruption and unauthorized access (Tahsien, Karimipour & Spachos, 2020; Mawgoud, Karadawy & Tawfik, 2019; Bhatia, Verma & Sharma, 2020; Vajar et al., 2021). The authentication, authorization, and privacy issues are currently discussed in IoT domains. However, some other security issues can also be addressed, such as secure data availability at the right time, resource authentication, integrity, and confidentiality (Das & Nene, 2017). The current state-of-the-art security models are not cover security in all the domains of IoT. Few of them are cover the IoMT security issues (Aljumaie et al., 2021; Raj & Madiajagan, 2021), and the remaining are focused on the IoV (Ali, Hassan & Saeed, 2021). Other domains exist in IoT, including IoH and IoT, which should also be secured from threats. In the current era, heavy models are implemented in IoT devices to deal with complex and dynamic attacks. All the IoT devices have less computing power and cannot tackle this heavy software to overcome the security issues. Different machine learning (Anbalagan et al., 2021) and deep learning techniques are implemented to maintain privacy in IoT. As the traditional deep learning models work with large data sets and for the training of that data, enormous computational power is required (Lv et al., 2020; Li et al., 2022). Therefore, a lightweight method has been required that covers the security issues in the domain of IoT and provides the authentic model to secure these devices from vulnerable attacks.

## Conclusion

We have followed the systematic approach to extract the machine learning and deep learning models in IoT devices. A systematic literature review analyzes the research trends in IoT for security. A query string is constructed and applied to different repositories to select the relevant publications. Proper inclusion-exclusion criteria and quality assessment are conducted to extract the related 50 articles from the repositories from 2016 to 2021.

Existing literature focused only on the single domain of IoT to implement security in all perspectives. However, in this SLR, by using the query string, bias selection of related articles is removed, and only those searched by the query string are selected. The result reveals that most papers are selected from journals and the top conferences. The selected papers discussed machine learning and deep learning techniques to implement security in IoT subdomains. In future work, machine learning and deep learning techniques will be implemented in other domains of IoT. Furthermore, various security parameters such as confidentiality, vulnerability, authentication, and privacy of data can be implemented to secure the IoT devices.

## Supplemental Information

10.7717/peerj-cs.1204/supp-1 Supplemental Information 1 Summary of the Studies

## Additional Information and Declarations

### Data Availability

The following information was supplied regarding data availability: There was no raw data in our literature review.
