# Supplementary material for: Machine learning and deep learning approaches in IoT
Source: PeerJ Comput Sci. 2023 Feb 6;9:e1204. doi: 10.7717/peerj-cs.1204 (PMC10280223; doi:10.7717/peerj-cs.1204)
Supplement: Supplemental Information 1 [file peerj-cs-09-1204-s001.docx]

Summary of the Studies

Paper 1

| Citation information | Reddy, D. K., Behera, H. S., Nayak, J., Vijayakumar, P., Naik, B., & Singh, P. K. (2021). Deep neural network based anomaly detection in Internet of Things network traffic tracking for the applications of future smart cities. *Transactions on Emerging Telecommunications Technologies*, *32*(7), e4121. |
| --- | --- |
| Cited by | 9 |
| Impact Factor | 1.2 |
| Venue | WEILY |
| Purpose of selection | The reason for selecting this paper is, as it is deal with the anomaly detection in the internet of things by using deep learning methods. |
| Subjects | Traffic tracking in smart cities applications |
| Methodology | Deep learning based framework is used with random neural network to classify anomaly. |
| Design and Analysis | Experimentation performance and evaluations on deep learning neural network architecture is being used to recognize seven categorical attacks. |
| Conclusion and Results | Deep neural network architecture performs well through noticeable improvement in most of the categorical attack. |
| Weakness | This paper not focus .anomaly or attack detection to defend networks from insecure IoT networks |
| Strength | Experimental results proof that deep neural network performs well to detect the attacks. |

Paper 2

| Citation information | Le, D. N., Parvathy, V. S., Gupta, D., Khanna, A., Rodrigues, J. J., & Shankar, K. (2021). IoT enabled depthwise separable convolution neural network with deep support vector machine for COVID-19 diagnosis and classification. *International journal of machine learning and cybernetics*, 1-14. |
| --- | --- |
| Cited by | 69 |
| Impact Factor | 3.72 |
| Venue | International journal of machine learning and cybernetics |
| Purpose of selection | This paper works on the applications of health care by using IoT technologies. |
| Subjects | Implement IoT technologies for healthcare diagnosis |
| Methodology | IoT enabled Depthwise Separable Convolution Neural Network (DWS-CNN) with Deep Support Vector Machine (DSVM) for COVID-19 Diagnosis and Classification |
| Design and Analysis | DSVM framework is employed to compute the binary and multiple class labels of COVID-19 |
| Conclusion and Results | DWS-CNN approach is sampled over the CXR image dataset and outcomes are proposed by means of diverse performance metrics. |
| Weakness | Healthcare diagnostics can also done by implementing the bio-inspired algorithms to attain maximum classification results. |
| Strength | DWS-CNN model attaining the maximum classification performance with the accuracy of 98.54% and 99.06% on binary and multiclass. |

Paper 3

| Citation information | Panda, M., Abd Allah, A. M., & Hassanien, A. E. (2021). Developing an Efficient Feature Engineering and Machine Learning Model for Detecting IoT-Botnet Cyber Attacks. *IEEE Access*, *9*, 91038-91052. |
| --- | --- |
| Cited by | 2 |
| Impact Factor | 3.7 |
| Venue | IEEE access |
| Purpose of selection | This paper deals the IoT botnet cyber-attacks by using machine learning algorithms.. |
| Subjects | Implement machine learning models on botnet cyber attacks |
| Methodology | UNSW-NB15, a new IoT-Botnet dataset to classify cyber-attacks. |
| Design and Analysis | Three recent machine learning (ML) methods JChaid, A2DE and HGC based classifiers are implemented. |
| Conclusion and Results | Scatter search-based DMLP classifier outperforms the other competing models. |
| Weakness | An efficient machine learning and deep learning model should be implemented with suitable feature engineering to detect and protect the network from vulnerabilities. |
| Strength | Low computational complexity with the least training time of 4.7 seconds & testing time of 0.61 seconds. |

Paper 4

| Citation information | Rouzbahani, H. M., Bahrami, A. H., & Karimipour, H. (2021). A Snapshot Ensemble Deep Neural Network Model for Attack Detection in Industrial Internet of Things. In *AI-Enabled Threat Detection and Security Analysis for Industrial IoT* (pp. 181-194). Springer, Cham. |
| --- | --- |
| Cited by | 1 |
| Impact Factor | 4.88 |
| Venue | Springer |
| Purpose of selection | This paper implement the deep neural network model for attack detection |
| Subjects | Industrial Internet of Things (IIoT) ongoing to emerge in different industry sectors |
| Methodology | A Snapshot Ensemble Deep Neural Network (SEDNN) has been utilized and evaluated. |
| Design and Analysis | Machine Learning- based technique is used in order to detect cyber-attacks on IIoT systems. |
| Conclusion and Results | Different metrics, including accuracy, precision, recall, and F1-score achieved are 87.42%, 93.77% and 90.48%. |
| Weakness | Results can also improve by implementing different other algorithms with different metrics. |
| Strength | The proposed model obtained an accuracy of 90.58% for cyber-attack detection. |

Paper 5

| Citation information | Alkahtani, H., & Aldhyani, T. H. (2021). Intrusion detection system to advance internet of things infrastructure-based deep learning algorithms. *Complexity*, *2021*. |
| --- | --- |
| Cited by | 1 |
| Impact Factor | 4.62 |
| Venue | Complexity |
| Purpose of selection | This paper covers the intrusion detection system by implementing deep learning algorithms. |
| Subjects | Robust framework system for detecting intrusions based on the IoT environment. |
| Methodology | To classify the intrusion three deep learning models are implemented such CNN, LSTM, and a hybrid CNN-LSTM models. |
| Design and Analysis | IoTID20 dataset attack was employed to develop the proposed system |
| Conclusion and Results | Performance attained on a new variable dataset, and the system will be implemented in our university IoT environment. |
| Weakness | This model can be implemented with other data set as the extension of used data set. |
| Strength | The proposed model effectively detects real-world attacks and is capable of enhancing the security of the IoT environment. |

| Citation information | Islam, N., Farhin, F., Sultana, I., Kaiser, M. S., Rahman, M. S., Mahmud, M., ... & Cho, G. H. (2021). Towards machine learning based intrusion detection in IoT networks. *Comput. Mater. Contin*, *69*, 1801-1821. |
| --- | --- |
| Cited by | 1 |
| Impact Factor | 3.77 |
| Venue | Computer, Materials and Continua |
| Purpose of selection | Intrusion detection in IoT using machine learning techniques. |
| Subjects | Node heterogeneity raised security concern in the IoT. |
| Methodology | Identify different types of IoT threats and shallow D, RF, SVM as well as deep machine learning DNN, DBN, LSTM, stacked LSTM, Bi-LSTM based intrusion detection systems in the IoT environment. |
| Design and Analysis | Five benchmark datasets such as NSL-KDD, IoTDevNet, DS2OS, IoTID20, and IoT Botnet are used to measure the performance of the system. |
| Conclusion and Results | Deep machine learning IDS outperforms shallow machine learning in detecting IoT attacks. |
| Weakness | Search is required on the problem based on real-time data and power time optimization. |
| Strength | The structure overcomes implementation problems of heavy DL techniques directly on low space IoT devices. |

Paper 6

| Citation information | Khan, M. A. (2021). HCRNNIDS: Hybrid Convolutional Recurrent Neural Network-Based Network Intrusion Detection System. *Processes*, *9*(5), 834. |
| --- | --- |
| Cited by | 8 |
| Impact Factor | 2.75 |
| Venue | Processes |
| Purpose of selection | Intrusion detection by implementing the advance deep learning algorithms. |
| Subjects | Hybrid convolutional recurrent neural network for intrusion detection |
| Methodology | Convolutional recurrent neural network (CRNN) is used to create a DL-based hybrid ID framework to predict and classifies malicious cyber-attacks in the network. |
| Design and Analysis | Recurrent neural network (RNN) captures temporal features to improve the ID system’s performance and prediction. |
| Conclusion and Results | The simulation outcomes prove that the proposed HCRNNIDS substantially outperforms current ID methodologies, attaining a high malicious attack detection rate accuracy up to 97.75% |
| Weakness | Different metrics should be used to measure the performance of the model. |
| Strength | Experiments were done on publicly available ID data CSE-CIC-DS2018 data. |

Paper 7

Paper 8

| Citation information | Rahman, M. A., Asyhari, A. T., Wen, O. W., Ajra, H., Ahmed, Y., & Anwar, F. (2021). Effective combining of feature selection techniques for machine learning-enabled IoT intrusion detection. *Multimedia Tools and Applications*, 1-19. |
| --- | --- |
| Cited by | 2 |
| Impact Factor | 2.75 |
| Venue | Multimedia Tools and Applications |
| Purpose of selection | IoT intrusion detection by feature selection techniques of machine learning |
| Subjects | Effective feature selection techniques to improve intrusion detection using machine learning methods |
| Methodology | SVM and decision tree to Naive Bayes for selecting high-ranked features and then artificial neural networks are implemented. |
| Design and Analysis | Proposed approach is based on a centralized intrusion detection system, which uses the deep feature abstraction, feature selection and classification to train the model for detecting the malicious and anomalous actions in the traffic. |
| Conclusion and Results | Effectiveness of the proposed method on Aegean Wi-Fi Intrusion Dataset, which achieves high detection accuracy of up to 99.95%. |
| Weakness | Method should be implemented to detect the more various attacks. |
| Strength | Achieve best results as compared to other machine learning algorithms. |

Paper 9

| Citation information | Sarker, I. H. (2021). CyberLearning: Effectiveness analysis of machine learning security modeling to detect cyber-anomalies and multi-attacks. *Internet of Things*, *14*, 100393. |
| --- | --- |
| Cited by | 6 |
| Impact Factor | 9.93 |
| Venue | Internet of Things |
| Purpose of selection | Machine learning security models to detect the cyber attacks |
| Subjects | Machine learning-based cyber security modeling with correlated-feature selection |
| Methodology | Binary [classification model](https://www.sciencedirect.com/topics/computer-science/classification-models) is implemented for [detecting anomalies](https://www.sciencedirect.com/topics/computer-science/detecting-anomaly)*,* and multi-class classification model is used for various types of cyber-attacks*.* |
| Design and Analysis | Employ the first ten machine learning algorithms and then implement the artificial neural network based security model based on multiple hidden layers. |
| Conclusion and Results | Effectiveness of these learning-based security models is examined by conducting a range of experiments utilizing the two most popular security datasets, UNSW-NB15 and NSL-KDD. |
| Weakness | This model can be implemented with more recent data with higher dimensions in IoT environment. |
| Strength | Empirical analysis and findings can be used as a reference guide in both academia and industry in the area of cyber security for effectively building a data-driven security modeling. |

Paper 10

| Citation information | Bedi, P., Mewada, S., Vatti, R. A., Singh, C., Dhindsa, K. S., Ponnusamy, M., & Sikarwar, R. (2021). Detection of attacks in IoT sensors networks using machine learning algorithm. *Microprocessors and Microsystems*, *82*, 103814. |
| --- | --- |
| Cited by | 3 |
| Impact Factor | 1.5 |
| Venue | Microprocessors and Microsystems |
| Purpose of selection | Implementation of machine learning algorithms to detect attacks in IoT sensors networks |
| Subjects | Detection of attacks in IoT sensor networks |
| Methodology | Machine learning (ML) module is implemented with foresee assault and abnormalities on the IoT frameworks. |
| Design and Analysis | ML algorithms have been used are Artificial Neural Network, Logistic Regression, Random Forest, Support Vector Machine, and Decision Tree. |
| Conclusion and Results | The assessment is measured by the different parameters such as f1 score, exactness, area, recollect, and precision under the ROC Curve. |
| Weakness | More in depth study of the organization of the whole structure is required. |
| Strength | Random Forest gives the best results as compared with the other ML algorithms. |

Paper 11

| Citation information | Latif, S., Zou, Z., Idrees, Z., & Ahmad, J. (2020). A novel attack detection scheme for the industrial internet of things using a lightweight random neural network. *IEEE Access*, *8*, 89337-89350. |
| --- | --- |
| Cited by | 24 |
| Impact Factor | 3.74 |
| Venue | IEEE Access |
| Purpose of selection | Attack detection in IoT using advance deep neural network |
| Subjects | Random neural network is used for attack detection in IoT. |
| Methodology | Novel lightweight random neural network (RaNN)-based prediction model |
| Design and Analysis | Performance of the RaNN-based prediction model is evaluated by parameters such as accuracy, precision, recall, and F1 score and compared with the traditional artificial neural network, support vector machine and decision tree. |
| Conclusion and Results | Evaluation results show that the proposed RaNN model achieves an accuracy of 99.20% for a learning rate of 0.01, with a prediction time of 34.51 milliseconds. |
| Weakness | This model should be implemented with the other new security related datasets. |
| Strength | Prediction time is fast as compared to other algorithms. |

Paper 12

| Citation information | Kasongo, S. M., & Sun, Y. (2020). A deep learning method with wrapper based feature extraction for wireless intrusion detection system. *Computers & Security*, *92*, 101752. |
| --- | --- |
| Cited by | 53 |
| Impact Factor | 1.64 |
| Venue | Computers & Security |
| Purpose of selection | Implemented deep learning method for intrusion detection |
| Subjects | Wrapper based feature extraction for wired intrusion detection system |
| Methodology | Feed-Forward Deep Neural Network (FFDNN) wireless IDS system using a Wrapper Based Feature Extraction Unit (WFEU) |
| Design and Analysis | The effectiveness and efficiency of the WFEU-FFDNN is studied based on the UNSW-NB15 and the AWID intrusion detection datasets. |
| Conclusion and Results | This approach achieved overall accuracies of 87.10% and 77.16% for the binary and multiclass classification schemes |
| Weakness | Investigate the detection rates of individual classes of the UNSW-NB15 and the AWID |
| Strength | Proposed WFEU-FFDNN has greater detection accuracy than other approaches. |

Paper 13

| Citation information | Khalifa, N. E. M., Taha, M. H. N., Manogaran, G., & Loey, M. (2020). A deep learning model and machine learning methods for the classification of potential coronavirus treatments on a single human cell. *Journal of Nanoparticle Research*, *22*(11), 1-13. |
| --- | --- |
| Cited by | 6 |
| Impact Factor | 2.00 |
| Venue | Journal of Nanoparticle Research |
| Purpose of selection | Implemented machine learning and deep learning methods for classification |
| Subjects | Potential coronavirus treatment on single human cell |
| Methodology | DCNN model |
| Design and Analysis | The proposed DCNN model consists of three convolutional layers, three ReLU layers, three pooling layers, and two fully connected layers. |
| Conclusion and Results | The dataset used is a subset of the publicly online datasets available on RxRx.ai, it gives the higher accuracy as compared to other machine learning models. |
| Weakness | Should be performed the same experiments with deep transfer models such as Alexnet and Resnet50 or even deeper neural networks to investigate its performance |
| Strength | The performance metrics strengthen the obtained results. |

Paper 14

| Citation information | HaddadPajouh, H., Dehghantanha, A., Khayami, R., & Choo, K. K. R. (2018). A deep recurrent neural network based approach for internet of things malware threat hunting. *Future Generation Computer Systems*, *85*, 88-96. |
| --- | --- |
| Cited by | 214 |
| Impact Factor | 7.18 |
| Venue | Future Generation Computer Systems |
| Purpose of selection | Implement the deep neural networks algorithms in IoT |
| Subjects | Deep recurrent neural network for IoT |
| Methodology | [Recurrent Neural Network](https://www.sciencedirect.com/topics/computer-science/recurrent-neural-network) (RNN) deep learning in detecting IoT malware |
| Design and Analysis | Uses an RNN model to analyze ARM-based IoT applications’ execution operation codes |
| Conclusion and Results | Evaluate the trained model using 100 new IoT [malware samples](https://www.sciencedirect.com/topics/computer-science/malware-sample) with three different Long Short Term Memory (LSTM) configurations. |
| Weakness | The proposed approach is not implemented in a real-world environment to identify both known malware and new malware. |
| Strength | LSTM method gives the best possible outcome. |

Paper 15

| Citation information | Shah, S. K., Tariq, Z., & Lee, Y. (2018, December). Audio iot analytics for home automation safety. In *2018 IEEE International Conference on Big Data (Big Data)* (pp. 5181-5186). IEEE. |
| --- | --- |
| Cited by | 11 |
| Impact Factor | 0.71 |
| Venue | IEEE International Conference on Big Data |
| Purpose of selection | Used the machine learning algorithms on IoT analytics |
| Subjects | Perform audio analytics through an audio Internet of Things. |
| Methodology | Shallow learning and deep learning based models are implemented for classification. |
| Design and Analysis | An automatic detection system is urgent for enforcing home safety and safe neighborhood. |
| Conclusion and Results | IoT system detects any suspicious sound, it generates an emergency notification to nearest emergency services for possible action to be taken. The classification is based on audio such as gunshots, explosion, glass breaking, screaming and siren. |
| Weakness | This model can be implemented with other real world audio data. |
| Strength | Convolutional Neural Network shows the best performance compared to other machine learning algorithms. |

Paper 16

| Citation information | Hasan, M., Islam, M. M., Zarif, M. I. I., & Hashem, M. M. A. (2019). Attack and anomaly detection in IoT sensors in IoT sites using machine learning approaches. *Internet of Things*, *7*, 100059. |
| --- | --- |
| Cited by | 210 |
| Impact Factor | 9.937 |
| Venue | Internet of Things |
| Purpose of selection | Machine learning algorithms are used to detect anomaly in IoT sites |
| Subjects | Attack and anomaly detection in IoT sensors |
| Methodology | The machine learning algorithms that are used: [Logistic Regression](https://www.sciencedirect.com/topics/computer-science/logistic-regression), [Support Vector Machine](https://www.sciencedirect.com/topics/computer-science/support-vector-machine), Decision Tree, Random Forest, and [Artificial Neural Network](https://www.sciencedirect.com/topics/computer-science/artificial-neural-network). |
| Design and Analysis | Performances of several [machine learning](https://www.sciencedirect.com/topics/computer-science/machine-learning) models have been compared to predict attacks and anomalies on the IoT systems accurately. |
| Conclusion and Results | E[valuation metrics](https://www.sciencedirect.com/topics/computer-science/evaluation-metric) used in the comparison of performance are accuracy, precision, recall, f1 score, and area under the Receiver Operating Characteristic Curve. |
| Weakness | This model can be implemented with other real world data. |
| Strength | Random Forest gives better results as compared to other models. |

Paper 17

| Citation information | Zhiqiang, L., Mohi-Ud-Din, G., Bing, L., Jianchao, L., Ye, Z., & Zhijun, L. (2019, August). Modeling Network Intrusion Detection System Using Feed-Forward Neural Network Using UNSW-NB15 Dataset. In *2019 IEEE 7th International Conference on Smart Energy Grid Engineering (SEGE)* (pp. 299-303). IEEE. |
| --- | --- |
| Cited by | 12 |
| Impact Factor | 153 H-index |
| Venue | IEEE 7th International Conference on Smart Energy Grid Engineering |
| Purpose of selection | Intrusion detection by implementing feed forward neural network |
| Subjects | Modeling network intrusion detection system |
| Methodology | A deep learning IDS is proposed using state of the art UNSW-NB15 dataset. |
| Design and Analysis | Select the optimal activation function and features and then testing on unseen data demonstrates high accuracy and lower false alarm rate. |
| Conclusion and Results | Deep Learning can effectively classify with high dimensionality and complex features. |
| Weakness | Not work on other dimensions of network intrusion. |
| Strength | Proposed classifier outperforms other machine learning models |

Paper 18

| Citation information | Doshi, R., Apthorpe, N., & Feamster, N. (2018, May). Machine learning ddos detection for consumer internet of things devices. In *2018 IEEE Security and Privacy Workshops (SPW)* (pp. 29-35). IEEE. |
| --- | --- |
| Cited by | 318 |
| Impact Factor | 2.04 |
| Venue | IEEE Security and Privacy Workshops |
| Purpose of selection | Machine learning algorithms implemented in IoT |
| Subjects | Ddos Detection for consumer devices |
| Methodology | IoT-specific network behaviors to inform feature selection to enhance the DDoS detection in IoT network traffic. |
| Design and Analysis | Botnets such as Mirai have used insecure consumer IoT devices to conduct distributed denial of service (DDoS) attacks on critical Internet infrastructure. |
| Conclusion and Results | Network middle boxes could automatically detect local IoT device sources of DDoS attacks using low-cost machine learning algorithms |
| Weakness | Not cover all the attacks. |
| Strength | Works on machine learning algorithms that provide the low cost to detect the attacks. |

| Citation information | Ullah and Q. H. Mahmoud, "Design and Development of a Deep Learning-Based Model for Anomaly Detection in IoT Networks," in IEEE Access, vol. 9, pp. 103906-103926, 2021, doi: 10.1109/ACCESS.2021.3094024 |
| --- | --- |
| Cited by |  |
| Impact Factor | 2.00 |
| Venue | Journal of Nanoparticle Research |
| Purpose of selection | implement the deep learning models in IoT |
| Subjects | Design of deep learning models for anomaly detection |
| Methodology | CNN model |
| Design and Analysis | Multiple techniques implemented for the detection of anomaly in IoT networks |
| Conclusion and Results | Accuracy of proposed convolutional neural network model is measured by using the BoT-IoT, IoT Network Intrusion, MQTT-IoT-IDS2020, and IoT-23 intrusion detection dataset |
| Weakness | Not implement in real time data set |
| Strength | Binary and multi classification implemented |

Paper 19

Paper 20

| Citation information | Smys, S., Basar, A., & Wang, H. (2020). Hybrid intrusion detection system for internet of Things (IoT). *Journal of ISMAC*, *2*(04), 190-199. |
| --- | --- |
| Cited by | 45 |
| Impact Factor | 0.42 |
| Venue | Journal of ISMAC |
| Purpose of selection | Intrusion detection system for IoT |
| Subjects | Intrusion detection system |
| Methodology | Hybrid CNN model |
| Design and Analysis | Intrusion detection system for IoT network and detect different types of attacks based on hybrid convolutional neural network model. |
| Conclusion and Results | Proposed research work is validated and compared with conventional machine learning and deep learning model. |
| Weakness | Not implement in real time data set |
| Strength | Proposed model is suitable for wide range of IoT applications. |

Paper 21

| Citation information | Mohanta, B. K., Jena, D., Mohapatra, N., Ramasubbareddy, S., & Rawal, B. S. (2021). Machine learning based accident prediction in secure iot enable transportation system. *Journal of Intelligent & Fuzzy Systems*, (Preprint), 1-13. |
| --- | --- |
| Cited by | 4 |
| Impact Factor | 1.85 |
| Venue | Journal of Intelligent & Fuzzy Systems |
| Purpose of selection | Machine learning used to secure IoT |
| Subjects | Accident prediction in IoT using machine learning algorithms |
| Methodology | Hybrid ML models implemented for classification |
| Design and Analysis | SVM, DT and RF |
| Conclusion and Results | Accuracy improved to early predict the accident in IoT devices |
| Weakness | Not implement deep learning models |
| Strength | Accuracy enhanced when compare with other methods. |

Paper 22

| Citation information | Dunn, C., Moustafa, N., & Turnbull, B. (2020). Robustness evaluations of sustainable machine learning models against data poisoning attacks in the internet of things. *Sustainability*, *12*(16), 6434. |
| --- | --- |
| Cited by | 7 |
| Impact Factor | 3.25 |
| Venue | Sustainability |
| Purpose of selection | Machine leaning models in IoT |
| Subjects | Robustness evaluation of sustainable machine learning models |
| Methodology | Modified machine learning models |
| Design and Analysis | The machine learning models have been evaluated using the ToN_IoT and UNSW NB-15 datasets, as they include a wide variety of recent legitimate and attack vector. |
| Conclusion and Results | Modified function is developed to manipulate legitimate input classes with different data rates. |
| Weakness | Not implement in real time data set. |
| Strength | Performance measured in terms of accuracy and detection rates. |

Paper 23

| Citation information | Susilo, B., & Sari, R. F. (2020). Intrusion detection in IoT networks using deep learning algorithm. *Information*, *11*(5), 279. |
| --- | --- |
| Cited by | 21 |
| Impact Factor | 0.34 |
| Venue | Information |
| Purpose of selection | IoT detection using deep learning algorithms |
| Subjects | Intrusion detection in IoT networks |
| Methodology | Modified Deep learning algorithm |
| Design and Analysis | CNN and ML is implemented on different data sets. |
| Conclusion and Results | CNN and random forest gives the best accuracy. |
| Weakness | Not implement in real time data set. |
| Strength | Performance enhanced as compared with other models. |

Paper 24

| Citation information | Kotenko, Igor, Igor Saenko, and Alexander Branitskiy. "Framework for mobile Internet of Things security monitoring based on big data processing and machine learning." *IEEE Access* 6 (2018): 72714-72723. |
| --- | --- |
| Cited by | 35 |
| Impact Factor | 3.7 |
| Venue | *IEEE Access* |
| Purpose of selection | The reason for selecting this paper is, as it is deal with the internet of things by using machine learning models. |
| Subjects | Combine the big data and machine learning models for Security monitoring. |
| Methodology | Dynamic Bayesian networks (DBNs) and deep learning neural networks (DLNNs) methods are used for anomaly detection. |
| Design and Analysis | DBNs are characterized by a graph-like structure, which defines a set of observable and unobservable random variables, changing over time in accordance with the transition model. DBNs are characterized by a graph-like structure, which defines a set of observable and unobservable random variables, changing over time in accordance with the transition model. |
| Conclusion and Results | Results of classifier operations are exposed to plurality voting, weighted voting and soft voting. |
| Weakness | Hadoop and Spark models can also implemented for the better deduction of anomaly. |
| Strength | Model allows detecting multi-step attacks and provides the ability to calculate the probability of how an observable event is anomalous. |

Paper 25

| Citation information | Holbrook, L., & Alamaniotis, M. (2019, November). Internet of things security analytics and solutions with deep learning. In *2019 IEEE 31st International Conference on Tools with Artificial Intelligence (ICTAI)* (pp. 178-185). IEEE. |
| --- | --- |
| Cited by | 2 |
| Impact Factor |  |
| Venue | *IEEE 31st International Conference on Tools with Artificial Intelligence (ICTAI)* |
| Purpose of selection | This paper deals with the deep learning models on internet of things for security analytics. |
| Subjects | Security analytics and solutions |
| Methodology | Deep learning models for critical security applications by utilizing snapshots of network traffic from nine real-world IoT devices. |
| Design and Analysis | Deep neural network provides the highest coefficient of determination compared to the other tested models. |
| Conclusion and Results | Support Vector Machines (SVM), Random Forest and Deep Neural Network (DNN) algorithms, obtained results exhibited that all three tested algorithms provided high accuracy. |
| Weakness | Not implement deduct all the anomalies |
| Strength | The DNN's learning autonomy feature allows omission of humans from the loop resulting in time efficient real-world algorithm. |

Paper 26

| Citation information | Khamparia, A., Gupta, D., de Albuquerque, V. H. C., Sangaiah, A. K., & Jhaveri, R. H. (2020). Internet of health things-driven deep learning system for detection and classification of cervical cells using transfer learning. *The Journal of Supercomputing*, 1-19. |
| --- | --- |
| Cited by | 43 |
| Impact Factor | 2.6 |
| Venue | The Journal of Supercomputing |
| Purpose of selection | This paper is included as it is works on deep learning systems for detection. |
| Subjects | Security analytics and solutions |
| Methodology | Deep learning models for critical security applications by utilizing snapshots of network traffic from nine real-world IoT devices. |
| Design and Analysis | Feature extraction from cervical images is performed using pre-trained CNN models like InceptionV3, VGG19, SqueezeNet and ResNet50, which are fed into dense and fattened layer for normal and abnormal cervical cells classification. |
| Conclusion and Results | Performance of the proposed IoHT frameworks is evaluated using standard Pap smear Herlev dataset. |
| Weakness | This model can be extend our system with the involvement of hybrid nature inspired techniques for the diagnosis of critical diseases like Alzheimer and aseptic encephalitis with advanced machine learning ensemble classifiers. |
| Strength | This model gives the minimum training and testing time. |

Paper 27

| Citation information | Sumathi, S., & Karthikeyan, N. (2021). Detection of distributed denial of service using deep learning neural network. *Journal of Ambient Intelligence and Humanized Computing*, *12*(6), 5943-5953. |
| --- | --- |
| Cited by | 7 |
| Impact Factor | 4.81 |
| Venue | *Journal of Ambient Intelligence and Humanized Computing* |
| Purpose of selection | This paper works on deep learning neural networks. |
| Subjects | Distributed denial of services |
| Methodology | DNN model |
| Design and Analysis | The performance metrics such as detection accuracy, cost per sample, average delay, packet loss, overhead, packet delivery ratio and throughput are used for the performance analysis. |
| Conclusion and Results | Simulation result observed that DNN Cost minimization algorithm provides better result in terms of high detection accuracy 99% with less false reduction. |
| Weakness | This model not deals with over fitting results. |
| Strength | This paper evaluates the network performance using deep learning neural network classifier with cost minimization strategy. |

Paper 28

| Citation information | Alam, F., Mehmood, R., Katib, I., & Albeshri, A. (2016). Analysis of eight data mining algorithms for smarter Internet of Things (IoT). Procedia Computer Science, 98, 437-442. |
| --- | --- |
| Cited by | 153 |
| Impact Factor | 2.09 |
| Venue | Procedia Computer Science |
| Purpose of selection | Included, this paper discuss the internet of things with data mining algorithms |
| Subjects | Analysis of data mining algorithms |
| Methodology | DLANNs |
| Design and Analysis | The deep learning artificial neural networks (DLANNs) model is used, which build a feed forward multi-layer artificial neural network (ANN) for modelling high-level data abstractions. |
| Conclusion and Results | IoT datasets show that C4.5 and C5.0 have better accuracy, are memory efficient and have relatively higher processing speeds. |
| Weakness | These algorithms are computationally expensive. |
| Strength | ANNs and DLANNs can provide highly accurate results. |

Paper 29

| Citation information | Sumathi, S., & Pugalendhi, G. K. (2021). Cognition based spam mail text analysis using combined approach of deep neural network classifier and random forest. *Journal of Ambient Intelligence and Humanized Computing*, *12*(6), 5721-5731. |
| --- | --- |
| Cited by | 6 |
| Impact Factor | 7.01 |
| Venue | Journal of Ambient Intelligence and Humanized Computing |
| Purpose of selection | Included, this paper used random forest and deep neural network algorithms |
| Subjects | Spam mail text analysis |
| Methodology | Random forest with deep neural networks |
| Design and Analysis | Deep Neural Network Classifier model (DNNs) is trained using back propagation algorithm in batch learning mode. The Gini measure is examined to rank the important features. |
| Conclusion and Results | An accuracy of 88.59% while considering the top ranked five features. |
| Weakness | Not compare with all the existing models |
| Strength | Classification rate of DNN is higher than compared to KNN and Support Vector Machine (SVM). |

Paper 30

| Citation information | Yavuz, F. Y., Ünal, D., & Gül, E. (2018). Deep learning for detection of routing attacks in the internet of things. *International Journal of Computational Intelligence Systems*, *12*(1), 39-58. |
| --- | --- |
| Cited by | 61 |
| Impact Factor | 1.7 |
| Venue | International Journal of Computational Intelligence Systems |
| Purpose of selection | Included, this paper discusses the deep learning with internet of things. |
| Subjects | Detection of routing attacks |
| Methodology | Highly scalable, deep-learning based attack detection. |
| Design and Analysis | Cooja IoT simulator has been utilized for generation of high-fidelity attack data, within IoT networks ranging from 10 to 1000 nodes. |
| Conclusion and Results | Detection of IoT routing attacks which are decreased rank, hello-flood and version number modification attacks, with high accuracy and precision. |
| Weakness | Not cover all the attacks. |
| Strength | Results get on self-collected dataset. |

Paper 31

| Citation information | Kasongo, S. M., & Sun, Y. (2020). A deep learning method with wrapper based feature extraction for wireless intrusion detection system. *Computers & Security*, *92*, 101752. |
| --- | --- |
| Cited by | 54 |
| Impact Factor | 5.7 |
| Venue | Computers & Security |
| Purpose of selection | Included, as it used the deep learning methods |
| Subjects | Feature extraction for wireless intrusion deduction system |
| Methodology | Feed-Forward Deep Neural Network (FFDNN) |
| Design and Analysis | The effectiveness and efficiency of the WFEU-FFDNN is studied based on the UNSW-NB15 and the AWID intrusion detection datasets. |
| Conclusion and Results | This approach achieved overall accuracies of 87.10% and 77.16% for the binary and multiclass classification schemes |
| Weakness | NA |
| Strength | Proposed WFEU-FFDNN has greater detection accuracy |

Paper 32

| Citation information | Zhou, Y., Han, M., Liu, L., He, J. S., & Wang, Y. (2018, April). Deep learning approach for cyberattack detection. In *IEEE INFOCOM 2018-IEEE Conference on Computer Communications Workshops (INFOCOM WKSHPS)* (pp. 262-267). IEEE. |
| --- | --- |
| Cited by | 55 |
| Impact Factor | 1.7 |
| Venue | Conference on Computer Communications Workshops (INFOCOM WKSHPS) |
| Purpose of selection | Included, as it used the deep learning methods for attack detection |
| Subjects | Internet intrusion in the IOT environment |
| Methodology | DFEL |
| Design and Analysis | Each layer contains some neurons with activation functions that are used to produce non-linear outputs. |
| Conclusion and Results | DFEL not only boosts classifiers' accuracy to predict cyberattack but also significantly reduce the detection time. |
| Weakness | Not work on real equipment to prevent cyberattacks. |
| Strength | Model balance the detection performance and speed |

Paper 33

| Citation information | Almiani, M., AbuGhazleh, A., Al-Rahayfeh, A., Atiewi, S., & Razaque, A. (2020). Deep recurrent neural network for IoT intrusion detection system. *Simulation Modelling Practice and Theory*, *101*, 102031. |
| --- | --- |
| Cited by | 57 |
| Impact Factor | 2.2 |
| Venue | Simulation Modelling Practice and Theory |
| Purpose of selection | Included, this paper cover the IoT with deep neural network |
| Subjects | IoT intrusion detection system |
| Methodology | Artificial full-automated [intrusion detection system](https://www.sciencedirect.com/topics/computer-science/intrusion-detection-system) |
| Design and Analysis | Proposed model uses multi-layered of [recurrent neural networks](https://www.sciencedirect.com/topics/engineering/recurrent-neural-network) designed to be implemented for Fog computing security that is very close to the end-users and [IoT devices](https://www.sciencedirect.com/topics/computer-science/internet-of-things-device). |
| Conclusion and Results | Proposed model using a balanced version of the challenging dataset: NSL-KDD. Mathew correlation and Cohen's [Kappa coefficients](https://www.sciencedirect.com/topics/computer-science/kappa-coefficient) features are used to measure the performance. |
| Weakness | Not work on real time dataset. |
| Strength | Model is more stable and robust as compare with others. |

Paper 34

| Citation information | Tuan, T. A., Long, H. V., Son, L. H., Kumar, R., Priyadarshini, I., & Son, N. T. K. (2020). Performance evaluation of Botnet DDoS attack detection using machine learning. *Evolutionary Intelligence*, *13*(2), 283-294. |
| --- | --- |
| Cited by | 49 |
| Impact Factor | 2.3 |
| Venue | Evolutionary Intelligence |
| Purpose of selection | Included, as it covers the machine learning models. |
| Subjects | IoT intrusion detection system |
| Methodology | Botnet DDoS |
| Design and Analysis | Machine learning methods SVM, ANN, NB, DT, and Unsupervised Learning are investigated for Accuracy, False Alarm Rate (FAR), Sensitivity, Specificity, False positive rate (FPR), AUC, and Matthews correlation coefficient (MCC) of datasets. |
| Conclusion and Results | The evaluation is done on the UNBS-NB 15 and KDD99 which are well-known publicity datasets for Botnet DDoS attack detection. |
| Weakness | Gives better accuracy on deep learning models. |
| Strength | Performance of KDD99 dataset has been experimentally shown to be better as compared to the UNBS-NB 15 dataset. |

Paper 35

| Citation information | Tuan, T. A., Long, H. V., Son, L. H., Kumar, R., Priyadarshini, I., & Son, N. T. K. (2020). Performance evaluation of Botnet DDoS attack detection using machine learning. *Evolutionary Intelligence*, *13*(2), 283-294. |
| --- | --- |
| Cited by | 49 |
| Impact Factor | 2.3 |
| Venue | Evolutionary Intelligence |
| Purpose of selection | Included, as it covers the machine learning models. |
| Subjects | IoT intrusion detection system |
| Methodology | Botnet DDoS |
| Design and Analysis | Machine learning methods SVM, ANN, NB, DT, and Unsupervised Learning are investigated for Accuracy, False Alarm Rate (FAR), Sensitivity, Specificity, False positive rate (FPR), AUC, and Matthews correlation coefficient (MCC) of datasets. |
| Conclusion and Results | The evaluation is done on the UNBS-NB 15 and KDD99 which are well-known publicity datasets for Botnet DDoS attack detection. |
| Weakness | Gives better accuracy on deep learning models. |
| Strength | Performance of KDD99 dataset has been experimentally shown to be better as compared to the UNBS-NB 15 dataset. |

Paper 36

| Citation information | Min, E., Long, J., Liu, Q., Cui, J., & Chen, W. (2018). TR-IDS: Anomaly-based intrusion detection through text-convolutional neural network and random forest. *Security and Communication Networks*, *2018*. |
| --- | --- |
| Cited by | 67 |
| Impact Factor | 1.79 |
| Venue | Security and Communication Networks |
| Purpose of selection | Included, as it covers the machine learning models for intrusion detection. |
| Subjects | Anomaly based intrusion detection |
| Methodology | TR-IDS |
| Design and Analysis | Word embedding and text-convolutional neural network (Text-CNN) is applied to extract effective information from payloads. The sophisticated random forest algorithm is performed on the combination of statistical features and payload features. |
| Conclusion and Results | The sophisticated random forest algorithm is implemented for the final classification. |
| Weakness | Not work well with other dataset. |
| Strength | Proposed method gives the best result |

Paper 37

| Citation information | Li, F., Li, Q., Zhang, J., Kou, J., Ye, J., Song, W., & Mantooth, H. A. (2020). Detection and diagnosis of data integrity attacks in solar farms based on multilayer long short-term memory network. IEEE Transactions on Power Electronics, 36(3), 2495-2498. |
| --- | --- |
| Cited by | 13 |
| Impact Factor | 8.02 |
| Venue | IEEE Transactions on Power Electronics |
| Purpose of selection | Included, as this paper detects the attacks. |
| Subjects | Detection of data integrity attacks |
| Methodology | Multilayer LSTM |
| Design and Analysis | MLSTM architecture not only remembers sequential information but also carries out more rigorous screening of time information. |
| Conclusion and Results | Compared with CNN, MLSTM achieves high detection accuracy even when the window size is 50 (0.05 s), and with longer analysis window length, MLSTM can even do better. |
| Weakness | This model should be implemented in real time. |
| Strength | MLSTM achieves the best performances in terms of all metrics, with only two layers. |

Paper 38

| Citation information | Manhas, J., & Kotwal, S. (2021). Implementation of Intrusion Detection System for Internet of Things Using Machine Learning Techniques. In Multimedia Security (pp. 217-237). Springer, Singapore. |
| --- | --- |
| Cited by | 1 |
| Impact Factor | 1.86 |
| Venue | Multimedia Security |
| Purpose of selection | Included, as this paper detects the attacks in IoT systems. |
| Subjects | Intrusion detection system for internet of things |
| Methodology | Machine learning |
| Design and Analysis | Different techniques of machine learning K-nearest neighbor, multilayer perceptron, decision tree, Naïve Bayes and support vector machine have been evaluated for implementation of IDS to classify network connections as normal or malicious. |
| Conclusion and Results | Accuracy, sensitivity, precision and F-score, have been taken to assess ability of machine learning techniques |
| Weakness | Not implement the deep learning models. |
| Strength | Decision tree is best classifier for IDS. |

Paper 39

| Citation information | Otoom, M., Otoum, N., Alzubaidi, M. A., Etoom, Y., & Banihani, R. (2020). An IoT-based framework for early identification and monitoring of COVID-19 cases. *Biomedical signal processing and control*, *62*, 102149. |
| --- | --- |
| Cited by | 70 |
| Impact Factor | 3.13 |
| Venue | Biomedical signal processing and control |
| Purpose of selection | Included, this paper covers the IoT based framework. |
| Subjects | Detection of Covid 19 cases |
| Methodology | SVM, neural network, naïve bayes, KNN, DT, and Decision stump, OneR and ZeroR. |
| Design and Analysis | Framework consists of five main components: Symptom Data Collection and Uploading, Quarantine/Isolation Center, Data Analysis Center, Health Physicians, and Cloud Infrastructure. |
| Conclusion and Results | An experiment was conducted to test these eight algorithms on a real COVID-19 symptom dataset, after selecting the relevant symptoms and achieves the best results. |
| Weakness | System should be implemented in real time. |
| Strength | Gives more than 90 % accuracy |

Paper 40

| Citation information | Vallathan, G., John, A., Thirumalai, C., Mohan, S., Srivastava, G., & Lin, J. C. W. (2021). Suspicious activity detection using deep learning in secure assisted living IoT environments. *The Journal of Supercomputing*, *77*(4), 3242-3260. |
| --- | --- |
| Cited by | 14 |
| Impact Factor | 2.6 |
| Venue | The Journal of Supercomputing |
| Purpose of selection | Included, this paper secure IoT environment |
| Subjects | Suspicious activity detection using deep learning |
| Methodology | Multi classifier |
| Design and Analysis | Abnormal activities are predicted using random forest differential evolution with kernel density (RFKD), and any abnormal activities that are detected cause signals to be sent to IoT devices via the MQTT protocol. |
| Conclusion and Results | The deep neural network is used to learn and train the data and kernel density is used clustering and prediction of data. |
| Weakness | Model can be implemented for tracking and detection of multiple anomalies detection in living environments. |
| Strength | The proposed work is in the dynamic nature of activity prediction. |

Paper 41

| Citation information | Atefinia, R., & Ahmadi, M. (2021). Network intrusion detection using multi-architectural modular deep neural network. *The Journal of Supercomputing*, *77*(4), 3571-3593. |
| --- | --- |
| Cited by | 9 |
| Impact Factor | 2.6 |
| Venue | The Journal of Supercomputing |
| Purpose of selection | Included, as this paper covers the deep learning algorithms |
| Subjects | Network intrusion detection |
| Methodology | Multi-architectural modular deep neural network model |
| Design and Analysis | Feed-forward module, a stack of restricted Boltzmann machine module, and two recurrent modules, which decrease the false-positive rate of anomaly based intrusion detection system. |
| Conclusion and Results | The experiments are performed using CSE-CIC-IDS2018 dataset, and final models can be used in an ID for generating alerts or preventing new attacks. |
| Weakness | Training time can be enhanced and used the custom dataset. |
| Strength | The proposed model gives the best result as it compared to others. |

Paper 42

| Citation information | Parra, G. D. L. T., Rad, P., Choo, K. K. R., & Beebe, N. (2020). Detecting Internet of Things attacks using distributed deep learning. *Journal of Network and Computer Applications*, *163*, 102662. |
| --- | --- |
| Cited by | 44 |
| Impact Factor | 6.2 |
| Venue | Journal of Network and Computer Applications |
| Purpose of selection | Included, as it covers the IoT attacks |
| Subjects | Detecting IoT attacks |
| Methodology | Cloud-based distributed deep learning |
| Design and Analysis | The model comprises two key security mechanisms a Distributed Convolutional Neural Network (DCNN) model and a cloud-based temporal Long-Short Term Memory (LSTM) network model. |
| Conclusion and Results | Proposed CNN model is capable of detecting phishing attacks with an accuracy of 94.3% and a F-1 score of 93.58%. Using the back-end LSTM model, the model detects Botnet attacks with an accuracy of 94.80% using all malicious data points in the used dataset. |
| Weakness | This can be implemented to detect other attacks. |
| Strength | The proposed approach is capable of detecting attacks, both at device and at the back-end level |

Paper 43

| Citation information | Ahmed, A. A., Jabbar, W. A., Sadiq, A. S., & Patel, H. (2020). Deep learning-based classification model for botnet attack detection. *Journal of Ambient Intelligence and Humanized Computing*, 1-10. |
| --- | --- |
| Cited by | 15 |
| Impact Factor | 4.81 |
| Venue | Journal of Ambient Intelligence and Humanized Computing |
| Purpose of selection | Included, as this paper use the deep learning based classification |
| Subjects | Classification model for botnet attack detection |
| Methodology | Feed forward propagation with ANN implementation |
| Design and Analysis | Proposed deep learning DNN and feed-forward backpropagation ANN technique to detect botnet attacks using the following steps, namely, dataset selection, feature extraction, data normalization, training, validation, and testing. |
| Conclusion and Results | NN designs reflect a decrease in their mean square error over time, but this decrease is likely to be reversed when the validation dataset begins to overfit the training data as it identifies random noise instead of underlying relationships. |
| Weakness | Not implemented to detect other malicious attacks |
| Strength | This paper gives the highest accuracy 99.6%. |

Paper 44

| Citation information | Al-Hawawreh, M., & Sitnikova, E. (2019, August). Industrial Internet of Things based ransomware detection using stacked variational neural network. In *Proceedings of the 3rd International Conference on Big Data and Internet of Things* (pp. 126-130). |
| --- | --- |
| Cited by | 8 |
| Impact Factor | 1.6 |
| Venue | Journal of Ambient Intelligence and Humanized Computing |
| Purpose of selection | Included, as this paper covers the industrial internet of things |
| Subjects | Ransomware attack detection using stacked variational neural network. |
| Methodology | stacked Variational Auto-Encoder (VAE) |
| Design and Analysis | Detection model based on the stacked Variational Auto-Encoder (VAE) with a fully connected neural network that is able to learn the latent structure of system activities and reveal the ransomware behavior. |
| Conclusion and Results | Data augmentation method based on VAE for generating new data that can be used in training a fully connected network in order to improve the generalized capabilities of the proposed detection model. |
| Weakness | Different other methods can be implemented to enhance the performance of the system. |
| Strength | Proposed model achieved considerable performance in detecting ransomware activities. |

Paper 45

| Citation information | Lin, P., Ye, K., & Xu, C. Z. (2019, June). Dynamic network anomaly detection system by using deep learning techniques. In *International conference on cloud computing* (pp. 161-176). Springer, Cham. |
| --- | --- |
| Cited by | 38 |
| Impact Factor | 2.53 |
| Venue | International conference on cloud computing |
| Purpose of selection | Included, as this paper includes the deep learning techniques. |
| Subjects | Dynamic network anomaly detection |
| Methodology | LSTM + AM |
| Design and Analysis | Long Short Term Memory (LSTM) to build a deep neural network model and add an Attention Mechanism (AM) to enhance the performance of the model. |
| Conclusion and Results | The classification accuracy of the model reaches 96.2%, which is higher than other machine learning algorithms. |
| Weakness | It deals limited class imbalance. |
| Strength | This model deals with the class imbalance problem. |

Paper 46

| Citation information | Mohammed, A. R., Mohammed, S. A., & Shirmohammadi, S. (2019, July). Machine learning and deep learning based traffic classification and prediction in software defined networking. In *2019 IEEE International Symposium on Measurements & Networking (M&N)* (pp. 1-6). IEEE. |
| --- | --- |
| Cited by | 21 |
| Impact Factor | 0.18 |
| Venue | IEEE International Symposium on Measurements & Networking |
| Purpose of selection | Included, as this paper covers the machine learning and deep learning techniques. |
| Subjects | Traffic classification and prediction |
| Methodology | SDN with deep learning |
| Design and Analysis | Traffic prediction will enable determining the possible congestion on the links before they lower the QoS & QoE and route the traffic to the less congested links. |
| Conclusion and Results | The evolution in the networking architecture has brought flexibility and extensibility. The decoupling of data and control planes has also made the network more prone to security issues. |
| Weakness | Routing optimization |
| Strength | ML and DL methods used for classification and prediction in SDNs. |

Paper 47

| Citation information | Souri, A., Ghafour, M. Y., Ahmed, A. M., Safara, F., Yamini, A., & Hoseyninezhad, M. (2020). A new machine learning-based healthcare monitoring model for student’s condition diagnosis in Internet of Things environment. *Soft Computing*, *24*, 17111-17121. |
| --- | --- |
| Cited by | 18 |
| Impact Factor | 3.51 |
| Venue | Soft Computing |
| Purpose of selection | Included, as this paper covers the machine learning techniques. |
| Subjects | Health care monitoring in IoT environment |
| Methodology | Support vector machine |
| Design and Analysis | Vital data are collected via IoT devices and data analysis is carried out through the machine learning methods for detecting the probable risks of student’s physiological and behavioral changes. |
| Conclusion and Results | The proposed model meets the efficiency and proper accuracy for detecting the students’ condition. The support vector machine has achieved the highest accuracy of 99.1% which is a promising result as compared to decision tree, random forest, and multilayer perceptron neural network algorithms as well. |
| Weakness | Not implement deep learning algorithms |
| Strength | SVM gives best result rather than other machine learning algorithms. |

Paper 48

| Citation information | Sharma, S., Dudeja, R. K., Aujla, G. S., Bali, R. S., & Kumar, N. (2020). DeTrAs: deep learning-based healthcare framework for IoT-based assistance of Alzheimer patients. *Neural Computing and Applications*, 1-13. |
| --- | --- |
| Cited by | 10 |
| Impact Factor | 4.77 |
| Venue | Neural Computing and Applications, |
| Purpose of selection | Included, as this paper covers the deep learning techniques for IoT based framework. |
| Subjects | Health care framework for IoT environment |
| Methodology | DeTrAs |
| Design and Analysis | DeTrAs has been evaluated using different datasets on the basis of accuracy, precision, recall, true-positive rate, false-positive rate, F-score and correctly classified instances. |
| Conclusion and Results | The correct classification instances percentage of RNN (DeTrAs) is 88.59%, which is 6% higher than nearest candidate, i.e., decision tree as well as the precision of RNN (DeTrAs) is higher than decision tree and Bayes Net on Daphnet dataset. |
| Weakness | Should be implemented Nash equilibrium. |
| Strength | DeTrAs outperforms the existing variants of its category due to deeper training using multiple layers of neural networks. |

Paper 49

| Citation information | Turgut, Z., Üstebay, S., Ali Aydın, M., Gürkaş Aydın, G. Z., & Sertbaş, A. (2019). Performance analysis of machine learning and deep learning classification methods for indoor localization in Internet of things environment. *Transactions on emerging telecommunications technologies*, *30*(9), e3705. |
| --- | --- |
| Cited by | 7 |
| Impact Factor | 2.6 |
| Venue | Transactions on emerging telecommunications technologies |
| Purpose of selection | Included, as it works in machine learning and deep learning models in IoT systems. |
| Subjects | Indoor localization in IoT environment |
| Methodology | RFKON_HIBRID |
| Design and Analysis | A reference signal map is obtained for the detection of the moving objects in the indoor areas that are in the IoT environment using the fingerprint method. |
| Conclusion and Results | The classification of the data passed through the PF with the SSAEs which is a DL method. |
| Weakness | Not implemented the signal map in self-organizing structure on the IoT environment. |
| Strength | Higher accuracy is achieved |

Paper 50

| Citation information | Kachavimath, A. V., & Narayan, D. G. (2021). A deep learning-based framework for distributed denial-of-service attacks detection in cloud environment. In *Advances in Computing and Network Communications* (pp. 605-618). Springer, Singapore. |
| --- | --- |
| Cited by | 2 |
| Impact Factor | 0.82 |
| Venue | Advances in Computing and Network Communications |
| Purpose of selection | Included, as it works in deep learning models. |
| Subjects | Denial of service attack detection in cloud environment |
| Methodology | DDoS attack detection by capturing pattern of sequences |
| Design and Analysis | DDoS attack detection was proposed by capturing different patterns of sequences from the captured traffic and analysis of the high-level features using deep learning and can be used with a high detection rate. |
| Conclusion and Results | Proposed methodology have demonstrated the better performance of long short-term memory (LSTM) approach with good accuracy compared to the convolutional neural network (CNN) and multilayer perceptron (MLP). |
| Weakness | Not cover all the attacks |
| Strength | Better results from the previous ones |

Paper 51

| Citation information | Xiao, F., Lin, Z., Sun, Y., & Ma, Y. (2019). Malware detection based on deep learning of behavior graphs. *Mathematical Problems in Engineering*, *2019*. |
| --- | --- |
| Cited by | 42 |
| Impact Factor | 1.3 |
| Venue | Mathematical Problems in Engineering |
| Purpose of selection | Included, as this paper discuss the deep learning techniques |
| Subjects | Malware detection based on deep learning |
| Methodology | novel behavior-based deep learning framework (BDLF) |
| Design and Analysis | Behavior graphs are constructed to provide efficient information of malware behaviors using extracted API calls. We then use a neural network-Stacked AutoEncoders (SAEs) for extracting high-level features from behavior graphs. |
| Conclusion and Results | Proposed BDLF can learn the semantics of higher-level malicious behaviors from behavior graphs and further increase the average detection precision by 1.5%. |
| Weakness | This model not be implemented for the classification of malware |
| Strength | Increased the average detection precision. |

Paper 52

| Citation information | Aminanto, M. E., Tanuwidjaja, H. C., Yoo, P. D., & Kim, K. (2017, September). Wi-Fi intrusion detection using weighted-feature selection for neural networks classifier. In *2017 International Workshop on Big Data and Information Security (IWBIS)* (pp. 99-104). IEEE. |
| --- | --- |
| Cited by | 17 |
| Impact Factor | 0.14 |
| Venue | International Workshop on Big Data and Information Security |
| Purpose of selection | Included, as this paper deals the intrusion detection |
| Subjects | Wi-fi intrusion detection using weighted selection |
| Methodology | weighted-based machine learning model |
| Design and Analysis | Wi-Fi networks since pervasive Internet-of-Things (IoT) devices create huge traffics and vulnerable at the same time. Test and validate the feasibility of the selected features using a common neural network. |
| Conclusion and Results | The experimental results demonstrate the effectiveness of the proposed model, achieving 99.72% F _1_ score, and prove that combining a weight-based feature selection method with a light machine-learning classifier improve the performance. |
| Weakness | This problem can be solved by deep learning models with better accuracy. |
| Strength | Increased the performance from the previous models. |

Paper 53

| Citation information | Priyadarshini, I., & Puri, V. (2021). A convolutional neural network (CNN) based ensemble model for exoplanet detection. *Earth Science Informatics*, *14*(2), 735-747. |
| --- | --- |
| Cited by | 6 |
| Impact Factor | 2.87 |
| Venue | Earth Science Informatics |
| Purpose of selection | Included, as it deals the deep learning |
| Subjects | Exoplanet detection using Convolutional neural network |
| Methodology | Ensemble-CNN model |
| Design and Analysis | The performance of the models has been evaluated using parameters like Accuracy, Precision, Sensitivity, and Specificity. |
| Conclusion and Results | The proposed Ensemble-CNN model performs relatively better for detecting exoplanets with an accuracy of 99.62%. |
| Weakness | Not cover all the areas |
| Strength | Increased the performance from the previous models. |

Paper 54

| Citation information | Saharkhizan, M., Azmoodeh, A., Dehghantanha, A., Choo, K. K. R., & Parizi, R. M. (2020). An ensemble of deep recurrent neural networks for detecting iot cyber attacks using network traffic. *IEEE Internet of Things Journal*, *7*(9), 8852-8859. |
| --- | --- |
| Cited by | 17 |
| Impact Factor | 9.93 |
| Venue | IEEE Internet of Things Journal |
| Purpose of selection | Included, as it deals the deep learning |
| Subjects | Detecting IoT cyber attcks |
| Methodology | LSTM + ensamble |
| Design and Analysis | Integrates a set of long short-term memory (LSTM) modules into an ensemble of detectors. These modules are then merged using a decision tree to arrive at an aggregated output at the final stage. |
| Conclusion and Results | The effectiveness of the model is using a real-world data set of Modbus network traffic and obtains an accuracy rate of over 99% in the detection of cyber attacks against IoT devices. |
| Weakness | Should be implemented more transparent on different IoT protocols |
| Strength | Used real time dataset |

Paper 55

| Citation information | Shahada, S. A. A., Hreiji, S. M., Atudu, S. I., & Shamsudheen, S. (2019). Multilayer Neural Network Based Fall Alert System Using IOT. *International Journal of MC Square Scientific Research*, *11*(4), 1-15. |
| --- | --- |
| Cited by | 22 |
| Impact Factor | 0.63 |
| Venue | International Journal of MC Square Scientific Research |
| Purpose of selection | Included, as it covers the IoT |
| Subjects | Fall alert system using IoT |
| Methodology | Multi-layer Neural Network |
| Design and Analysis | A smart setup to monitor human behavior through accelerometer, pulse sensor and GSM.  An alert system is used to the personal care assistant, to monitor the data using different learning methods. |
| Conclusion and Results | Multi-layer Neural Network technique data are collected from the sensors, then processed and passed to the server in the form of an alert through the buzzer, SMS, email or voice message. |
| Weakness | Cover limited areas |
| Strength | This model gives the accuracy more than the other models |

Paper 56

| Citation information | Oniani, S., Marques, G., Barnovi, S., Pires, I. M., & Bhoi, A. K. (2021). Artificial Intelligence for Internet of Things and Enhanced Medical Systems. In *Bio-inspired Neurocomputing* (pp. 43-59). Springer, Singapore. |
| --- | --- |
| Cited by | 16 |
| Impact Factor | 3.9 |
| Venue | Bio-inspired Neurocomputing |
| Purpose of selection | Included, as this paper deals the IoT |
| Subjects | Artificial intelligence for Internet of things |
| Methodology | AI + ML algorithms |
| Design and Analysis | Robotic surgery systems such as Transoral Robotic Surgery and Automated Endoscopic System for Optimal Positioning lead to several advantages as these methods provide less aggressive treatments and provide better results in terms of blood loss and faster recovery. |
| Conclusion and Results | Internet of medical things addresses numerous health conditions such a vital biophysical parameters supervision, diabetes, and medical decision-making support methods. |
| Weakness | Not implement deep learning algorithms |
| Strength | Compare different ML models with the extension of AI |

Paper 57

| Citation information | Sumaiya Thaseen, I., Saira Banu, J., Lavanya, K., Rukunuddin Ghalib, M., & Abhishek, K. (2021). An integrated intrusion detection system using correlation‐based attribute selection and artificial neural network. *Transactions on Emerging Telecommunications Technologies*, *32*(2), e4014. |
| --- | --- |
| Cited by | 17 |
| Impact Factor | 2.63 |
| Venue | Transactions on Emerging Telecommunications Technologies |
| Purpose of selection | Included, as this paper cover the intrusion detection using ANN |
| Subjects | AN integrated intrusion detection system |
| Methodology | Neural netwrok |
| Design and Analysis | A correlation-based feature selection integrated with neural network for identifying anomalies. |
| Conclusion and Results | Experimental analysis performed on NSL-KDD and UNSW-NB datasets, which are benchmark datasets of intrusion detection with current attacks. The proposed model is superior in terms of accuracy, sensitivity, and specificity in comparison with some of the state-of-the-art techniques. |
| Weakness | IDS Not implement in real word |
| Strength | A secure integrated network management can be achieved which is error-free and thereby improving performance. |
